# Microbiome function underpins the efficacy of a fiber-supplemented dietary intervention in dogs with chronic large bowel diarrhea

**DOI:** 10.1186/s12917-022-03315-3

**Published:** 2022-06-24

**Authors:** Dale A. Fritsch, Matthew I. Jackson, Susan M. Wernimont, Geoffrey K. Feld, Jennifer M. MacLeay, John J. Brejda, Chun-Yen Cochrane, Kathy L. Gross

**Affiliations:** 1 Global Clinical Nutrition and Claims, Hill’s Pet Nutrition, Inc., P.O. Box 1658, 1035 43rd St., Topeka, KS 66601-1658 USA; 2 Hill’s Pet Nutrition, Inc., 1035 NE 43rd St., Topeka, KS USA; 3 Metabolon, Inc., 617 Davis Dr, Morrisville, NC USA; 4 Geocyte, Dublin, OH USA; 5 AKC Canine Health Foundation, Inc., Raleigh, NC USA; 6 Alpha Statistical Consulting, Lincoln, NE USA

**Keywords:** Chronic diarrhea, Dietary intervention, Canines, Fiber, Metabolomics, Microbiome, Metagenomics, Postbiotics, Tryptophan metabolism, Endocannabinoids

## Abstract

**Background:**

Chronic large bowel diarrhea is a common occurrence in pet dogs. While nutritional intervention is considered the primary therapy, the metabolic and gut microfloral effects of fiber and polyphenol-enriched therapeutic foods are poorly understood.

**Methods:**

This prospective clinical study enrolled 31 adult dogs from private veterinary practices with chronic, active large bowel diarrhea. Enrolled dogs received a complete and balanced dry therapeutic food containing a proprietary fiber bundle for 56 days. Metagenomic and metabolomic profiling were performed on fecal samples at Days 1, 2, 3, 14, 28, and 56; metabolomic analysis was conducted on serum samples taken at Days 1, 2, 3, 28, and 56.

**Results:**

The dietary intervention improved clinical signs and had a clear effect on the gut microfloral metabolic output of canines with chronic diarrhea, shifting gut metabolism from a predominantly proteolytic to saccharolytic fermentative state. Microbial metabolism of tryptophan to beneficial indole postbiotics and the conversion of plant-derived phenolics into bioavailable postbiotics were observed. The intervention altered the endocannabinoid, polyunsaturated fatty acid, and sphingolipid profiles, suggesting a modulation in gastrointestinal inflammation. Changes in membrane phospholipid and collagen signatures were indicative of improved gut function and possible alleviation of the pathophysiology related to chronic diarrhea.

**Conclusions:**

In dogs with chronic diarrhea, feeding specific dietary fibers increased gut saccharolysis and bioavailable phenolic and indole-related compounds, while suppressing putrefaction. These changes were associated with improved markers of gut inflammation and stool quality.

**Supplementary Information:**

The online version contains supplementary material available at 10.1186/s12917-022-03315-3.

## Background

Chronic colitis and other inflammatory enteropathies are the most common causes of chronic large bowel diarrhea in dogs [1, 2]. The pathophysiology of chronic diarrhea is multifactorial, resulting from a complex interplay within the intestinal microenvironment between bacteria, dietary compounds, the immune system, environmental triggers, and host genetics [3–5].

Nutritional interventions are considered preferred treatments for chronic large bowel diarrhea, partly due to the accumulating evidence demonstrating the contribution of gut microbial dysbiosis to this condition [6, 7]. The gastrointestinal (GI) microbiome represents an ecosystem of organisms that play important symbiotic roles in digestion, metabolism, nutrient absorption, and immunomodulation [8]. Pet foods with specific fiber formulations that activate and nourish the canine GI microbiome and encourage selective microbial fermentation of the fiber in the gut may be particularly useful among dogs with chronic diarrhea.

The gut microbiome produces nutrients that nourish the intestinal epithelium, facilitate nutrient metabolism, prime the immune system, and protect hosts from enteropathogens [8, 9]. Bacteria-derived metabolites, or postbiotics, represent energy sources, regulate gastrointestinal motility, have anti-inflammatory properties, and strengthen the intestinal barrier [10–14]. In dogs, postbiotics that affect GI health include short-chain fatty acids (SCFAs), phenolic compounds of plant origin, transformed phenolic compounds, secondary bile acids, and proteolytic products, including indoles [8, 15]. In dogs with chronic enteropathy (CE) and colitis, reduced community diversity or prominence of individual bacterial pathobiont species consisting of an unhealthy microflora, or dysbiosis, has been observed [16–18]. Dysbiosis can result from infection, the use of antibiotics, or protein overconsumption and can lead to the production of undesirable metabolic end products, including pro-inflammatory metabolites, and reduced energy for enterocytes, as well as adverse changes in water balance, mucus production, immune function and nutrient absorption [8, 19–21]. Therefore, for these dogs, dietary changes that support desirable GI bacteria and promote the production of beneficial postbiotics, including the addition of fiber blends, may provide the necessary substrates for healthy intestinal microbial growth and reduce the clinical signs of canine large bowel diarrhea.

A novel fiber blend has been developed that consists of both soluble and insoluble fibers specifically chosen for their pre- and postbiotic activity, water-holding and stool-bulking capacity, and fiber-bound plant components [15]. In previous studies, a dietary intervention with this novel fiber blend improved stool scores, increased fecal saccharolytic products, and increased fecal antioxidant and anti-inflammatory metabolites compared with control foods in cats and dogs [15, 22, 23]. This intervention also shifted the composition of and metabolism by the GI microbiome toward saccharolytic fermentation, decreased putrefactive metabolites, and improved stool quality compared to the control food.

We conducted a prospective study evaluating the impact of a fiber-supplemented dietary intervention known to contain antioxidant and polyphenol compounds on parameters of GI health in dogs actively experiencing large bowel diarrhea. An analysis of the clinical results from the study demonstrated that the fiber-supplemented dietary intervention rapidly improved stool quality and resolved clinical signs of chronic diarrhea in the study population [24]. Pet owners also reported improvement in their dog’s stooling behaviors and quality of life. The objective of the present study was to evaluate the impact of this fiber-supplemented dietary intervention on the bacterial communities and metabolites of dogs with chronic large bowel diarrhea by conducting metabolomic and metagenomic analyses.

## Results

### Demographics and study disposition

The demographics and the disposition of the dogs included in the study have been previously reported [24]. In brief, 39 dogs with chronic diarrhea from 12 veterinary clinics in the United States were recruited and 31 were enrolled in the study. Eight dogs were excluded due to the presence of intestinal parasites (*n* = 4), age (*n* = 3), or the use of antibiotics (*n* = 1). The mean age of the dogs included in the study was 5.3 years (range, 1─10 years). Five dogs failed to successfully transition onto the study food during the first week of the study and were dismissed. Four other dogs discontinued the study: one due to low food intake (Day 23) and three for reasons unrelated to study food, including the presence of a rectal polyp (Day 12), the presence of a previously undiagnosed heart condition (Day 6), and owner noncompliance (Day 6). A total of 22 dogs completed the 8-week study according to protocol. The 31 dogs included in the intent-to-treat population began the fiber-supplemented food after initial clinical and fecal characterization at baseline (Day 1).

### Stool composition

Clinical results for the fecal scores in this study have been previously reported [24]. Briefly, significant improvements in diarrhea occurred within 1 day of the initiation of the dietary intervention and complete resolution was observed in 59% of dogs by Day 28 and in 68% of dogs by Day 56, with no cases of recurrence. Mean stool firmness, assessed on a 5-point scale, significantly increased from a grade of 2.6 ± 0.2 on Day 1 to 3.8 ± 0.2 on Day 2 (*P* < 0.0001). Within 24 hours of switching to the test food, the food had significantly reduced moisture content of the feces compared to Day 1, and significant reductions were evident at every visit through 4 weeks (Table 1, Supplementary Table 1). Total fecal ash levels were significantly reduced on Days 3, 28, and 56, relative to Day 1. Calcium, magnesium, phosphorus, and potassium all showed significant and durable reductions from baseline at one or more visits (Supplementary Table 1).

**Table 1:** Fecal composition from canines consuming intervention food

	Day 1	Day 2	Day 3	Day 14	Day 28	Day 56
n	27	28	30	23	22	22
Moisture, %	70.1 (1.2)	67.6 (0.8)*	68.1 (0.8)*	66.4 (1.2)**	67.3 (0.8)*	68.6 (0.7)
pH	5.9 (0.3)	6.1 (0.1)	6.1 (0.1)	5.9 (0.1)	5.9 (0.1)	5.9 (0.0)
Ash, %	6.6 (0.6)	5.8 (0.5)	4.5 (0.2)**	6.4 (1.0)	4.9 (0.2)**	4.8 (0.2)**

*P < 0.05; **P < 0.01 compared to Day 1. Data are presented as means (SE)

### Microbiome analysis

No statistically significant changes in alpha or beta diversity in the fecal samples were observed. PICRUSt-predicted functional Kyoto Encyclopedia of Genes and Genomes (KEGG) Orthology (KO) compositions of the arginine, benzoate, butyrate, phenylalanine, propionate, tryptophan, and tyrosine pathways also showed no significant changes (results not shown). However, analysis of individual KOs revealed that certain KOs significantly changed relative abundances from Day 1 to Days 2 and 3, which coincided with the observed clinical improvement of diarrhea. This indicated that the dietary intervention induced significant alterations in abundances of enzymes responsible for gut bacterial metabolism of the amino acids phenylalanine and tryptophan, as well as the SCFA butyrate. These KO results with their corresponding enzyme commission (EC) numbers, and metabolic super- and sub-pathways are listed in Table 2 (other significant results for Days 14, 28 and 56 are listed in Supplementary Table 2). Overall, 15 KOs/EC numbers were identified and these were associated with 53 metabolic sub-pathways, two human disease pathways, and one cellular process pathway. They accounted for 13, 12, 11, and 9 entries in the carbohydrate, xenobiotic (including “terpenoids and polyketides”), amino acid (including “other amino acids”), and lipid metabolic super-pathways, respectively.

**Table 2:** Significant Day 2 and 3 effects in selected PICRUSt-predicted functional pathways

Study day	Value*	FDR corrected *P* value	KEGG orthology (KO) group	Enzyme Commision (EC) number	Pathway	Specific pathways
2	−1.351	0.0109	K00023	acetoacetyl-CoA reductase [EC:1.1.1.36]	Carbohydrate	Glyoxylate and dicarboxylate, butanoate
2	−0.717	0.0386	K00169	pyruvate ferredoxin oxidoreductase, alpha subunit [EC:1.2.7.1]	Carbohydrate	Butanoate, propanoate, citrate cycle (TCA), glycolysis/gluconeogenesis, pyruvate
					Energy	Carbon fixation pathways in prokaryotes, methane
					Xenobiotics	Nitrotoluene degradation
2	−0.734	0.0359	K00170	pyruvate ferredoxin oxidoreductase, beta subunit [EC:1.2.7.1]	Carbohydrate	Butanoate, propanoate, citrate cycle (TCA), glycolysis/gluconeogenesis, pyruvate
					Energy	Carbon fixation pathways in prokaryotes, methane
					Xenobiotics	Nitrotoluene degradation
2	−0.717	0.0386	K00172	pyruvate ferredoxin oxidoreductase, gamma subunit [EC:1.2.7.1]	Carbohydrate	Butanoate, Propanoate, citrate cycle (TCA), glycolysis/gluconeogenesis, pyruvate
					Energy	Carbon fixation pathways in prokaryotes, methane
					Xenobiotics	Nitrotoluene degradation
2	−0.631	0.0273	K01426	amidase [EC:3.5.1.4]	Amino acids	Phenylalanine, arginine and proline, tryptophan
					Other amino acids	Cyanoamino acid
					Xenobiotics	Styrene and aminobenzoate degradation
2	−1.164	0.0314	K01617	4-oxalocrotonate decarboxylase [EC:4.1.1.77]	Xenobiotics	Xylene, dioxin, and benzoate degradation
2	−1.247	0.0109	K01907	acetoacetyl-CoA synthetase [EC:6.2.1.16]	Carbohydrate	Butanoate
					Lipid	Lipid biosynthesis proteins
2	−1.144	0.0034	K01908	propionyl-CoA synthetase [EC:6.2.1.17]	Carbohydrate	Propanoate
					Lipid	Lipid biosynthesis proteins
2	0.126	0.0301	K01963	acetyl-CoA carboxylase carboxyl transferase subunit beta [EC:6.4.1.2]	Carbohydrate	Propanoate, pyruvate
					Lipid	Fatty acid biosynthesis
					Energy	Carbon fixation pathways in prokaryotes
					Terpenoids and polyketides	Tetracycline biosynthesis
2	0.151	0.0175	K04072	acetaldehyde dehydrogenase/alcohol dehydrogenase [EC:1.2.1.10 1.1.1.1]	Carbohydrate	Butanoate, pyruvate, glycolysis/gluconeogenesis
					Lipid	Fatty acid biosynthesis
					Xenobiotics	Xylene, dioxin, naphthalene, benzoate, chloroalkane and chloroalkene degradation
					Amino acids	Tyrosine
2	−1.302	0.0204	K07516	3-hydroxyacyl-CoA dehydrogenase [EC:1.1.1.35]	Lipid	Fatty acid biosynthesis
					Energy	Carbon fixation pathways in prokaryotes
3	−1.415	0.0071	K00023	acetoacetyl-CoA reductase [EC:1.1.1.36]	Carbohydrate	Butanoate, glyoxylate and dicarboxylate
3	−1.017	0.0343	K00451	homogentisate 1,2-dioxygenase [EC:1.13.11.5]	Amino acids	Tyrosine
					Xenobiotics	Styrene degradation
3	−0.709	0.0107	K01426	amidase [EC:3.5.1.4]	Amino acids	Phenylalanine, arginine and proline, tryptophan
					Other amino acids	Cyanoamino acid
					Xenobiotics	Styrene and aminobenzoate degradation
3	−0.728	0.042	K01580	glutamate decarboxylase [EC:4.1.1.15]	Carbohydrate	Butanoate
					Amino acids	Alanine, aspartate and glutamate metabolism
					Other amino acids	beta-alanine, taurine and hypotaurine
					Metabolic diseases^h^	Type 1 diabetes mellitus
3	−0.833	0.0264	K01692	enoyl-CoA hydratase [EC:4.2.1.17]	Carbohydrate	Butanoate, propanoate
					Lipid	Fatty acid biosynthesis
					Amino acids	Tryptophan and/lysine, valine, leucine and isoleucine degradation
					Other amino acids	beta-alanine
					Terpenoids and Polyketides	Geraniol, limonene and pinene degradation
					Xenobiotics	Aminobenzoate, benzoate, and caprolactam degradation
3	−1.218	0.0134	K01907	acetoacetyl-CoA synthetase [EC:6.2.1.16]	Carbohydrate	Butanoate
					Lipid	Fatty acid biosynthesis
3	0.122	0.0377	K01963	acetyl-CoA carboxylase carboxyl transferase subunit beta [EC:6.4.1.2]	Carbohydrate	Propanoate
					Energy	Carbon fixation pathways in prokaryotes, methane
					Lipid	Fatty acid biosynthesis
					Terpenoids and polyketides	Tetracycline biosynthesis
3	−0.667	0.0386	K03781	catalase [EC:1.11.1.6]	Neurodegenerative^h^	Amyotrophic lateral sclerosis
					Transport and catabolism^c^	Peroxisome
					Amino acids	Tryptophan
					Energy	Methane
3	−1.219	0.0331	K07516	3-hydroxyacyl-CoA dehydrogenase [EC:1.1.1.35]	Energy	Carbon fixation pathways in prokaryotes, methane
					Lipid	Fatty acid biosynthesis

*Values are the estimated log ratios of relative pathway abundances between the respective Day and Day 1. All pathways were designated as metabolic pathways unless otherwise designated by superscript: c = cellular processes, h = human disease

### Fecal and serum metabolomics

To assess the biochemical contributions underpinning the efficacy of the dietary intervention, global untargeted metabolomic profiling was performed on canine stool (Days 1, 2, 3, 14, 28, and 56) and serum (Days 1, 2, 3, 28, and 56). Multivariate analysis of variation (MANOVA) for metabolite pathways in feces and serum are presented in Table 3 and Table 4, respectively, and all univariate data, including pathway assignments, are presented in Supplementary Table 3 and Supplementary Table 4, respectively. Serum metabolomics data were considered to delineate possible overlapping biological factors (e.g., nutrient absorption vs availability) and to evaluate the systemic impact of the intervention. *P* values reported in the text are for the highest order polynomial term that was statistically significant (*P* < 0.05).

**Table 3:** Fecal metabolomics summary

Metabolite Classification		MANOVA ***P***-value	Change vs Baseline
Alkaloids		0.01^b^	Mixed
Amino acids		0.0008^a^	Decreased
Benzoate metabolism		0.03^b^	Mixed
Carbohydrates		0.002^a^	Mixed
Collagen metabolism		0.02^a^	Decreased
Dipeptides		0.001^a^	Decreased
Endocannabinoids		0.02^a^	Decreased
Free Fatty Acids	Polyunsaturated n3	0.02^b^	Mixed
	Polyunsaturated n6	0.02^a^	Decreased
	Saturated & Monounsaturated	0.06^a^	Decreased
Hemoglobin metabolism		0.06^a^	Decreased
Linolenate metabolism		0.03^c^	Increased
Monoacylglycerols		0.04^a^	Mixed
Phenolics		0.08^a^	Mixed
Phospholipids	Lysophospholipids	ns	
	Phosphatidylcholines (PC)	0.001^b^	Decreased
	Phosphatidylethanolamines (PE)	ns	
	Phospholipid metabolism	ns	
Polyamines		0.03^a^	Decreased
Postbiotics		0.02^b^	Mixed
Primary bile acids		0.06^a^	Decreased
Secondary bile acids		ns	
Sphingolipids	All	0.08^a^	Decreased
	Ceramides	0.09^a^	Decreased
	Dihydroceramides	0.01^a^	Decreased
	Dihydrosphingomyelins	0.04^a^	Decreased
	Hexosylceramides (HCER)	0.02^a^	Decreased
	Sphingolipid synthesis	0.02^a^	Decreased
	Sphingosines	ns	
Sphingomyelins		0.009^a^	Decreased
Terpenoids		0.0004^a^	Increased
Tocopherol metabolism		0.04^b^	Mixed
Tryptophan metabolism	Indole pathway	0.0003^a^	Mixed
	Kynurenine pathway	ns	
	Serotonin pathway	0.048^a^	Decreased

^a^Linear trend over days, ^b^quadratic trend over days, ^c^cubic trend over days, ns = not significant; mixed directionality varied depending upon the metabolite in the group

**Table 4:** Serum metabolomics summary

Metabolite Classification		MANOVA ***P***-value	Change vs Baseline
Amino acids		< 0.0001^c^	Mixed
Carbohydrates		0.002^c^	Mixed
Collagen metabolism		< 0.0001^c^	Mixed
Free fatty acids	Eicosanoids	0.01^a^	Mixed
	Polyunsaturated n3	< 0.0001^a^	Increased
	Polyunsaturated n6	< 0.0001^a^	Decreased
	Saturated & monounsaturated	< 0.0001^a^	Mixed
Phospholipids	Lysophospholipids	< 0.0001^c^	Mixed
	Phosphatidylcholines (PC)	< 0.0001^c^	Mixed
	Phosphatidylethanolamines (PE)	0.005^c^	Mixed
	Phosphatidylinositol (PI)	< 0.0001^a^	Mixed
Sphingolipids	All	< 0.0001^a^	Decreased
	Ceramides	0.01^c^	Decreased
	Dihydroceramides	0.03^a^	Mixed
	Dihydrosphingomyelins	< 0.0001^a^	Mixed
	Hexosylceramides (HCER)	0.007^a^	Decreased
	Lactosylceramides (LCER)	0.02^c^	Mixed
	Sphingolipid synthesis	ns	
	Sphingosines	ns	
Sphingomyelins		0.03^c^	Mixed
Tocopherol metabolism		0.002^c^	Mixed
Tryptophan indole pathway		0.02^c^	Increased

^a^Linear trend over days, ^b^quadratic trend over days, ^c^cubic trend over days, ns = not significant, mixed directionality varied depending upon the metabolites in the group

### Assessment of putrefactive and fermentative metabolism

**Fig. 1:**
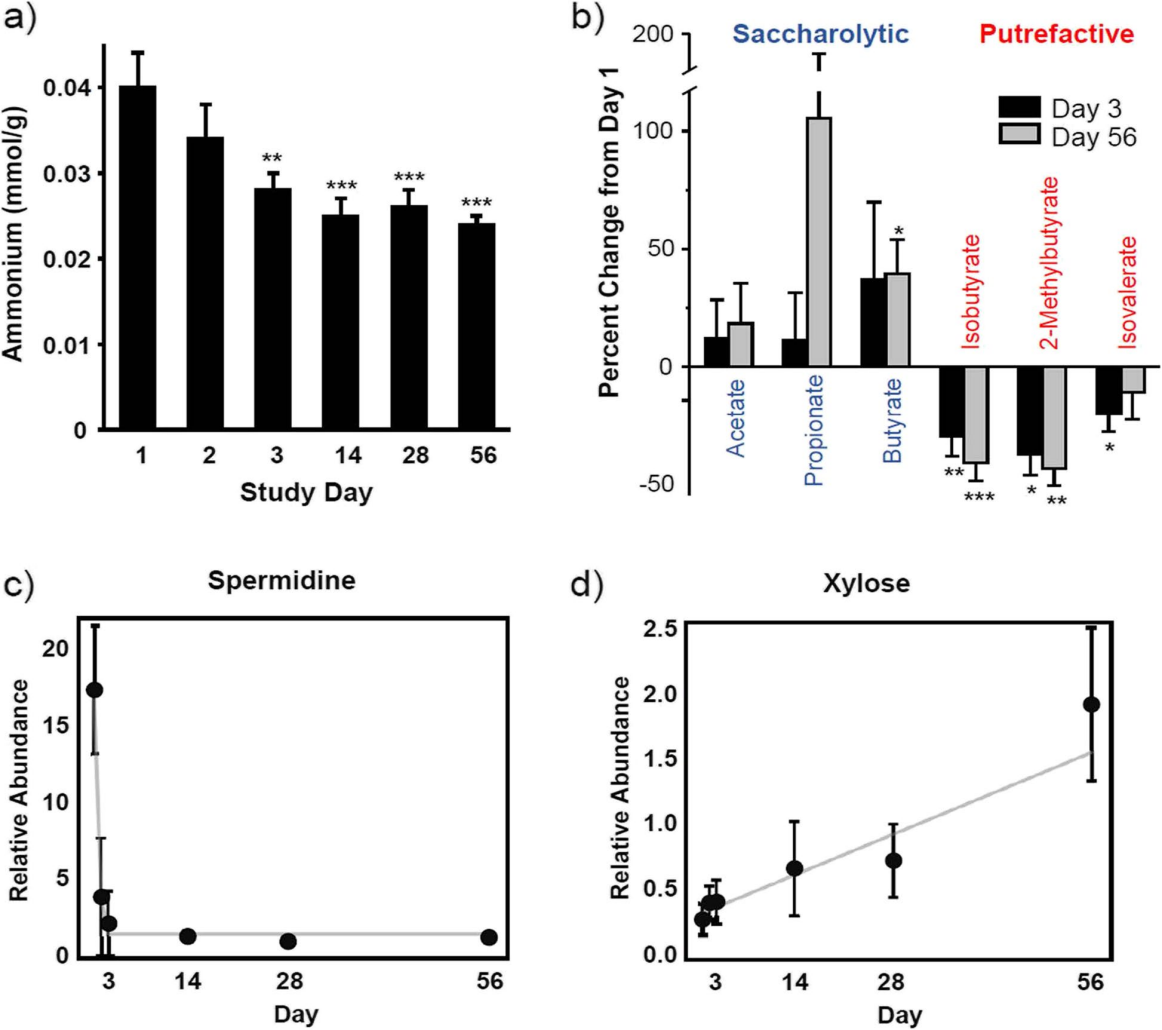
The dietary intervention’s impact on fermentative metabolism, including fecal ammonium concentrations (**A**) and percent changes in short chain fatty acids from baseline measurements at Days 3 and 56 (**B**), and representative fecal metabolomics relative abundance measurements plotted over time for the polyamine spermidine (**C**) and the monosaccharide xylose (**D**). Gray lines indicate a statistical model of the data; for spermidine this is a quadratic fit plateauing at Day 3, while xylose was fitted to a linear model. Error bars herein represent the standard error of measurements taken from group sizes ranging from 11 to 27 (Day 1), 8–28 (Day 2), 10–30 (Day 3), 3–23 (Day 14), 6–22 (Day 28), and 14–22 (Day 56). *P < 0.05, **P < 0.01; ***P < 0.001

To determine the impact of the dietary intervention on bacterial putrefactive metabolism, levels of several proteolytic metabolites in stool were assessed. Fecal ammonium concentration decreased from baseline at Day 3, and the reduced levels were durable through Day 56 (Fig. 1a). Fecal branched SCFAs also decreased at every study visit and at the end of the study (Fig. 1b). Isobutyrate and 2-methylbutyrate levels were significantly reduced at Days 3─56 and isovalerate reached significance at Days 3─4 and 28. The relative fecal abundances of the 20 proteogenic amino acids, plus taurine, were significantly impacted in a multivariate manner over the course of the intervention (*P* = 0.0008). Among the amino acids, 18 reached significance (only cysteine, histidine, and arginine did not, see Supplementary Table 3) and most decreased. Additionally, multivariate analysis of 19 identified dipeptides indicated these intermediate products of proteolysis were also significantly impacted by the dietary intervention (*P* = 0.001), among which six decreased significantly.

In serum, several individual amino acids significantly increased, and as a class they were significantly altered, as assessed by MANOVA (*P* < 0.0001, Table 4). Amino acids that significantly increased in serum and decreased in stool included asparagine, glycine, leucine, lysine, proline, taurine, threonine, tryptophan, and tyrosine (Supplementary Tables 3–4).

Multivariate analysis of the fecal polyamines, their acetylated derivatives, and reaction intermediates indicated this metabolic pathway was significantly reduced upon dietary intervention (*P* = 0.03, Table 3). The polyamine precursor lysine (*P* = 0.048), as well as polyamines cadaverine (*P* = 0.02), and N-acetyl cadaverine (*P* = 0.006), were significantly reduced (Supplementary Table 3). Levels of agmatine (*P* = 0.01), putrescine (*P* = 0.02) and spermidine (*P* = 0.0002) showed significant reductions from baseline upon dietary intervention, as shown in Fig. 1c, as did most of their mono- and diacetylated derivatives, including the bi-product 5′-methylthioadenosine (MTA; *P =* 0.03), and the oxidative end-product carboxyethyl-GABA (*P* = 0.02).

To assess whether the high-fiber food affected saccharolytic processes, the fecal metabolic products of fermentation were analyzed. Levels of the linear SCFA acetate and propionate did not significantly change from baseline (Fig. 1b), while butyrate significantly increased at Day 56 (*P* = 0.01), relative to baseline. The C5 and C6 fatty acids valerate (*P* = 0.003) and caproate (*P* = 0.03) decreased significantly over the course of the study (Supplementary Table 3). Monosaccharides in stool increased significantly as a class when assessed by MANOVA (*P* = 0.002, Table 3). Specific plant-derived monosaccharides that increased included xylose (*P* = 0.006), as shown in Fig. 1d, arabinose (*P* = 0.01), mannose (*P* = 0.03), and ribulose/xylulose (*P* = 0.004), the latter of which were isobar and not resolved. Monosaccharides that decreased over the study included ribose (*P* = 0.01), galactitol (*P* = 0.02), erythrose (*P* = 0.01), glucuronate (*P* = 0.02), and mannitol/sorbitol (*P* = 0.06).

MANOVA analysis demonstrated that carbohydrate metabolites as a class were also significantly altered in serum upon intervention (*P* = 0.002, Table 4). Notably, monosaccharides that significantly changed in both stool and circulation did so in opposite directions amongst the sample matrices, including mannitol/sorbitol (*P* < 0.05) and ribose (*P* = 0.04), which increased in serum, as well as mannose (*P* = 0.004), which decreased in serum (Supplementary Table 4). The other altered serum carbohydrates either did not change appreciably or were not measured in feces. Additionally, circulating levels of the linear SCFA butyrate increased significantly over the study (*P* = 0.005).

### Analysis of tryptophan metabolism

**Fig. 2:**
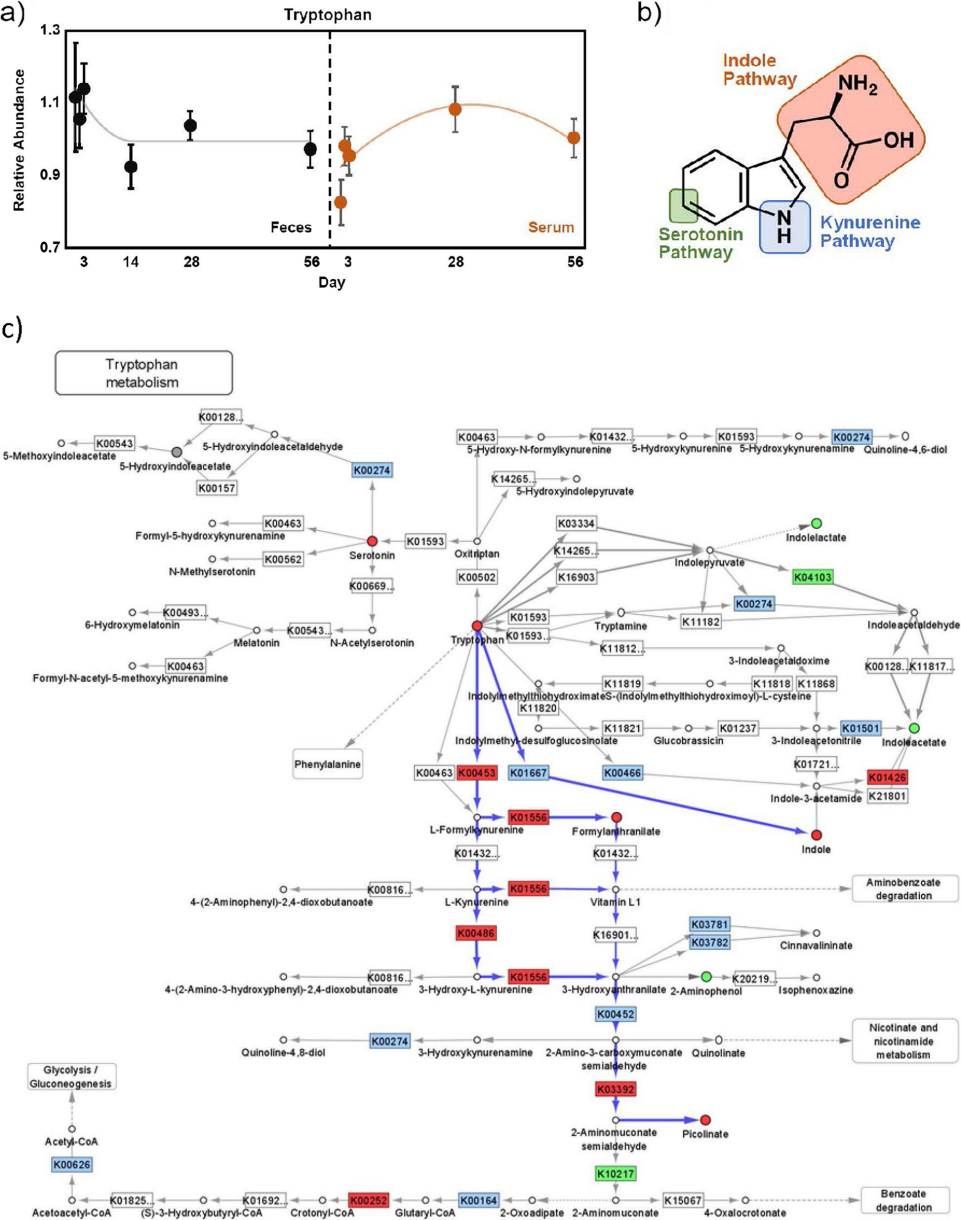
Effect of a therapeutic dog food on tryptophan metabolism. (**A**) Relative abundance measurements plotted over time for tryptophan in feces (black circles, left) and serum (orange circles, right). Gray line indicates a statistical model consisting of a quadratic fit plateauing at day 14. Orange line indicates a quadratic fit. (**B**) Tryptophan pathway schematic indicating where on the molecule the first reaction step acts upon to generate catabolites in the indicated pathway—kynurenine through pyrrole ring cleavage, serotonin through 5′ hydroxylation, and indole through chemistries leaving the side chain intact. (**C**) Simplified projection of fecal metabolite and inferred functional metagenomic measurements on the KEGG tryptophan pathway map (KO00380) [25–27]. Significantly increased and decreased enzymatic functions and metabolites are depicted in green and red, respectively, while observed but unchanged functions and metabolites are rendered grey. Likely catabolic paths discussed in the text are indicated with blue arrows

Given that our microbiome pathway analysis suggested that the intervention significantly impacted microbial tryptophan metabolism and altered both fecal and serum tryptophan levels (Fig. 2a), we stratified the tryptophan-related metabolites measured in this study into its three primary catabolic pathways—indole, serotonin, and kynurenine (Fig. 2b). The fiber intervention significantly altered the indole pathway as a group in feces (*P* = 0.0003), but the directionality of the change varied by metabolite. Indole (*P* = 0.03), indolin-2-one (*P* = 0.002), and the renal toxin 3-indoxyl sulfate (3-IS, *P* = 0.03) significantly decreased. Conversely, significant increases in indoleacetate (*P* = 0.02) and two of its downstream metabolites, indole-3-carboxylate (*P* = 0.001) and 2-oxindole-3-acetate (*P* = 0.008) were observed. Consumption of the high-fiber food significantly reduced the serotonin pathway (*P* = 0.048), whereas levels of serotonin decreased (*P* = 0.04) and its catabolic product 5-hydroxyindoleacetate increased (*P* = 0.03). Finally, the study food did not affect the kynurenine pathway as a group, as assessed by MANOVA. Downstream catabolites were differentially altered, as 2-aminophenol increased (*P* = 0.01) and picolinate decreased (*P* = 0.009), while levels of the upstream metabolite N-formylanthranilic acid were decreased (*P* = 0.04).

An analysis of the systemic impact of changes in fecal indole metabolism indicated that the intervention significantly altered measured tryptophan indole metabolites in serum (*P* = 0.02, Table 4). Among those metabolites that increased in both serum and feces were indoleacetate (*P* = 0.005) and 2-oxindole-3-acetate (*P* = 0.01). Compounds that increased in circulation but were unchanged in stool included indolepropionate (*P* = 0.006), indolelactate (*P* = 0.03), and indoleacrylate (*P* = 0.002), while indoleacetylalanine (*P* = 0.004), 3-indoleglyoxylic acid (*P* = 0.02), and 5-hydroxyindole sulfate (*P* = 0.045) were altered in serum but were not observed in stool (Supplementary Tables 3–4).

To better understand the mechanisms underlying our observed changes in metabolite measurements and inferred metagenomic functions, we integrated projections of these changes into a simplified representation of the KEGG tryptophan pathway map (KO00380), which is depicted in Fig. 2c. Inspection of the resulting map reveals several candidate pathways consistent with the experimental observations. Reduced tryptophan combined with reduced abundance and/or activity of tryptophan 2,3-dioxygenase (K00453) and kynureninase (K01556) resulted in an observed reduction of formylanthranilate and an additional reduction of aminocarboxymuconate-semialdehyde decarboxylase (K03392) activity, ultimately resulting in reduced picolinate. While not all intermediate activities and metabolites were observed or changed, there are several alternate paths consistent with observed functional changes (e.g., via L-kynurenine, 3-hydroxy-L-kynurenine) that could contribute to the reduction in picolinate.

The observed reduction in indole can be directly explained by the reduction in tryptophan while tryptophanase activity was not significantly changed over the course of the study (Fig. 2c). The significant reduction in serotonin is not supported by any experimental observations in this map. Increases in indoleacetate were consistent with the significant increase in indolepyruvate decarboxylase (K04103) abundance, although changes in intermediates to further support the activity of this pathway in feces were not observed. Moreover, increases in indolelactate and 2-aminophenol, while statistically significant, cannot be explained by changes in enzymatic activity mapped to the tryptophan pathway alone, but could result from increases in substrate and/or activity in reactions not mapped to tryptophan metabolism (e.g., benzoate degradation).

### Analysis of postbiotics derived from plant components

The canine fecal metabolome contained several compounds derived from plant fibers that we classified into phenolic compounds, alkaloids, and terpenoids for multivariate analysis (Table 3). We observed that the fiber intervention significantly impacted several phenolic compounds within 24 hours. Polyphenols that significantly increased included chrysoeriol (*P* = 0.02), diosmetin (*P* = 0.0002), sinensetin (*P* = 0.03), tangeritin (*P* = 0.03), and tetramethyl-o-scutellarin (*P* = 0.01), all of which are *n*-O-methyl flavones highly enriched in citrus fruits (Supplementary Table 3). Conversely, univariate analysis showed significant reductions among the likely soy-derived isoflavones daidzein (*P* = 0.02), genistein (*P* = 0.03), and glycitein (*P* = 0.02) in canine stool with the diet intervention. Additionally, citrus-sourced terpenoids limonin (*P* = 0.0008) and nomilin (*P* = 0.046) significantly increased over the study.

Levels of plant terpenoids and tocopherols in feces were also significantly altered in the study (*P* = 0.0004 and *P* = 0.04, respectively, Table 3). The phytosterols beta-sitosterol (*P* = 0.046), stigmastadienone (*P* = 0.02) and ergosterol (*P* = 0.002) increased in canine feces over the course of the study, while lanosterol (*P* = 0.007) decreased (Supplementary Table 3). Increases were reported for two thirds of the identified carotene diol species, while the intervention significantly increased fecal levels of the triterpenoid oleanolate (*P* = 0.003). Circulating tocopherols were also significantly altered in serum (*P* = 0.002, Table 4). Modified tocopherol metabolites tended to change in the same direction in both serum and stool; alpha-tocopherol acetate and alpha- carboxyethyl hydroxychroman (CEHC) sulfate both increased while gamma-tocopherol/beta-tocopherol decreased, the latter of which were isobar and could not be resolved. The primary vitamin E metabolite, alpha-tocopherol, was unchanged in stool, yet it significantly increased in the circulation (*P* < 0.0001).

**Fig. 3:**
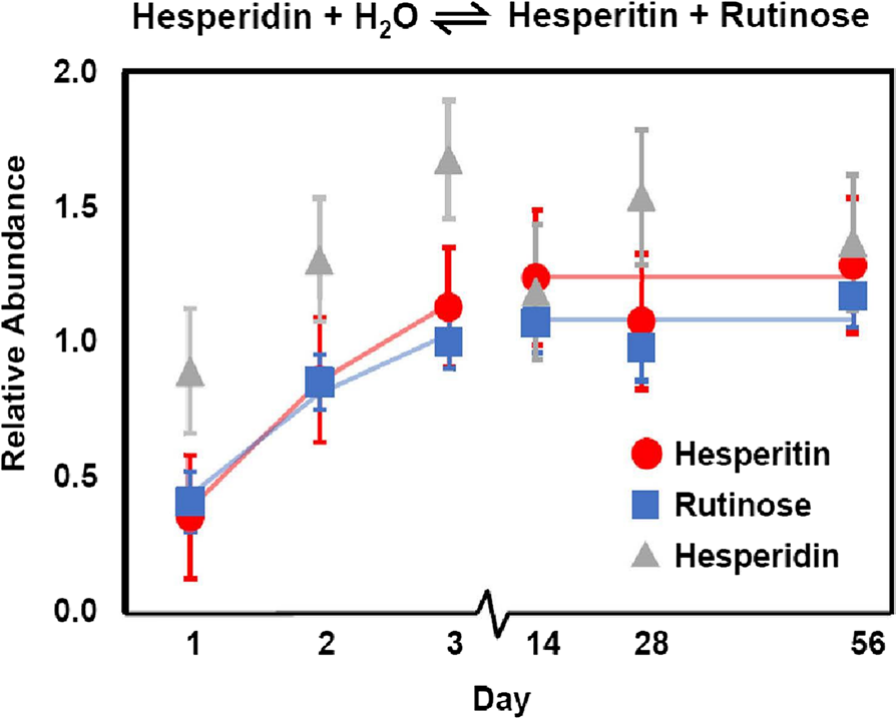
Reaction mechanism and relative abundance measurements plotted over time for the related plant-derived compounds hesperidin, hesperitin, and rutinose. The time axis is not to scale to improve the clarity of the first three measurements. Lines depict quadratic fits for hesperitin and rutinose plateauing at day 14. For hesperidin, no statistical models significantly represented the data

To identify the potential functional consequences of the fiber intervention on gut microbiota, we also evaluated changes in bioactive compounds that likely resulted from microfloral metabolism on plant parent biochemicals, also known as postbiotics. Multivariate analysis indicated the dietary intervention significantly affected the class of the 18 compounds identified as postbiotics (*P* = 0.02, Table 3), among which there were seven compounds either significantly increased or decreased. While the fiber intervention had no appreciable effect on hesperidin levels, significant increases were observed for hesperitin (*P* = 0.0001) and rutinose (*P* = 0.005), as shown in Fig. 3 (Supplementary Table 3). Narirutin, the 7-O-rutinoside of naringenin, was also unchanged, while levels of naringenin, the 7-O-rutinoside of ponciretin, significantly increased (*P* = 0.02) with the dietary intervention. Ponciretin significantly increased (*P* < 0.0001), and while poncirin was not measured, neoponcirin was unchanged.

Finally, the impact of the dietary intervention on bioactive phytoestrogens was considered. The fiber intervention resulted in significant reductions of fecal equol (*P* = 0.03) and enterolactone (*P* = 0.04), while leaving levels of enterodiol unaffected (Supplementary Table 3). Concomitantly, secoisolariciresinol diglucoside trended towards an increase (*p* = 0.08), while levels of both secoisolariciresinol (*P* = 0.04) and matairesinol (*P* = 0.03) were significantly increased in the stool of canines receiving the food.

### Dietary treatment effects on fatty acid metabolism

**Fig. 4:**
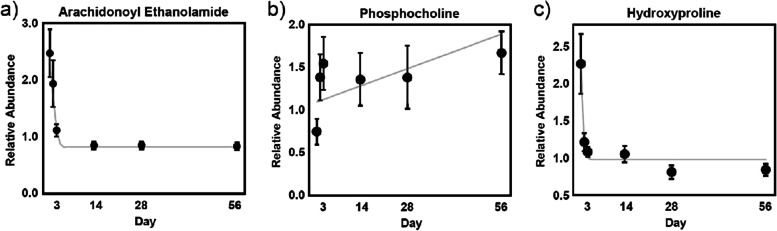
Relative abundance measurement plots over time in feces for representative metabolites in GI-related pathways, including the endocannabinoid arachidonoyl ethanolamide (**A**), with the gray line indicating a quadratic fit plateauing at Day 5; the phospholipid metabolic marker phosphocholine (**B**), with the gray line indicating a linear fit; and the collagen marker hydroxyproline (**C**), with the gray line indicating a quadratic fit plateauing at Day 4

The ligands of the endocannabinoid receptor system, N-acyl amides of ethanolamide and the serine-linked fatty acids, showed significant reductions in feces with the dietary intervention (*P* = 0.02, Table 3). Notably, reductions were evident among all identified ethanolamide-linked endocannabinoids by univariate analysis, typically within 1 day, regardless of fatty acid length and saturation (Supplementary Table 3). A representative abundance-over-time plot is presented for the well-studied human endocannabinoid arachidonoyl ethanolamide (*P* = 0.0001) in Fig. 4a. Exceptions included the N-acyl ethanolamides comprising the essential dietary polyunsaturated fatty acids (PUFAs) linoleate (linoleoyl ethanolamide, *P* = 0.028) and linolenate (linolenoyl ethanolamide, *P* = 0.011), which did not initially decrease within 24 hours (data not shown). By contrast, no changes in N-palmitoylserine or the glycerol-containing endocannabinoid 2-linoleoylglycerol (18:2) were observed. Additionally, levels of 2-oleoylglycerol (18:1) showed a trend toward increase, which was consistent with the directionality of monoacylglycerol species that did appreciably change after initiation of the dietary intervention (Supplementary Table 3). Thus, consumption of the high-fiber food resulted in reduced fecal levels of ethanolamine-containing endocannabinoids.

Substantial and statistically significant changes in both n3 and n6 PUFAs were observed in the stool following the dietary intervention (Table 3). Consistent with an overall increase in n3 PUFAs (*P* = 0.02), most of the individual n3 fatty acids increased over the study, with eicosapentaenoate (EPA; 20:5n3, *P* = 0.02) and stearidonate (18:4n3, *P* = 0.002) reaching significance (Supplementary Table 3). Docosatrienoate (22:3n3) was an exception, which significantly decreased (*P* = 0.009). The essential free fatty acid linolenate also significantly increased (*P* = 0.0003), although the isomerism (n3 vs n6) could not be resolved; therefore, we additionally analyzed all linolenate-containing species. This group significantly increased (*P* = 0.03), as shown in Table 3, including significant univariate increases in monoacylglycerol 1-linolenoylglycerol and several linolenate-containing diacylglycerols (Supplementary Table 3). Considering these observed increases and the inclusion of linolenate-rich flaxseed in the intervention fiber bundle [24], we assumed that the measured compound was, indeed, n3 linolenate and therefore included it in the n3 PUFA multivariate analysis. In contrast, fecal n6 PUFAs as a class were significantly reduced over the study (*P* = 0.02). Dihomolinoleate (20:2n6), docosatrienoate (22:2n6), and adrenate (22:4n6) all decreased significantly, while arachidonate (20:4n6) showed a non-significant reduction with the intervention.

Strikingly similar trends were observed for circulating serum PUFAs. Both n3 (*P* < 0.001) and n6 (*P* < 0.0001) derivatives were significantly altered in the same direction as in feces upon dietary intervention (Tables 3 and 4). All measured free n3 fatty acids significantly increased, except for docosatrienoate, which was unchanged and was the only n3 fatty acid that decreased in feces (Supplementary Table 4). Reductions in the serum n6 fatty acids docosadienoate, docosatrienoate, adrenate, and docosapentaenoate were observed. Intriguingly, circulating eicosanoids were also impacted by the dietary intervention (*P* = 0.01). Those derived from n6 (arachidonate) metabolism largely decreased upon intervention, whereas the reduction in 12-hydroxyeicosatetraenoic acid (12-HETE) reached significance (*P* = 0.004), while reductions in thromboxane B2, prostaglandin F2alpha, and 12-hydroxyheptadecatrienoic acid (12-HHTrE) trended toward significance.

We differentiated sphingomyelins from other sphingolipids and further classified the latter compounds into dihydrosphingomyelins, ceramides, dihydroceramides, hexosylceramides (HCERs), lactosylceramides (LCERs; detected in serum only), sphingosines, and sphingolipid synthesis biochemicals. Remarkably, multivariate analysis indicated that the dietary intervention statistically suppressed the fecal levels of sphingomyelins (*P* = 0.009), while non-significant reductions in other sphingolipids were also noted (*P* = 0.08), as shown in Table 3. Univariate analysis revealed that every identified fecal sphingolipid metabolite showed reductions from baseline over the 56-day study that were either statistically significant or trended towards statistical significance (Supplementary Table 3).

The high-fiber food also significantly affected circulating sphingomyelins as a class (*P* < 0.028) and the additional sphingolipids (*P* < 0.0001), including ceramides (*P* = 0.01), dihydroceramides (*P* = 0.03), HCERs (*P* = 0.007), dihydrosphingomyelins (*P* < 0.0001), and LCERs (*P* = 0.02) (Table 4, Supplementary Table 4). Univariate analysis revealed only four specific sphingolipid species out of 25 detected that reached significance, including three dihydrosphingomyelins and one LCER. While few changes in levels of individual sphingolipid species reached significance, significant changes in 28 detected sphingomyelins were observed, among which 6 significantly decreased and 14 significantly increased. Thus, two thirds of the measured choline-containing membrane-associated sphingolipids (i.e., sphingomyelins plus dihydrosphingomyelins) were significantly altered with the intervention.

### Evidence for restructuring of host GI tract

We analyzed fecal membrane lipid metabolism for clues suggestive of mucosal layer restructuring. Multivariate analysis (Table 3) indicated significant reductions in phosphatidylcholine (PC) lipids from baseline with the fiber intervention (*P* = 0.001). Of the 11 unique PC species identified, 7 significantly decreased, 3 were unchanged, and only 1-palmitoyl-2-docosahexaenoyl-GPC (16:0/22:6) significantly increased (*P* = 0.02, Supplementary Table 3). Such trends were not evident for phosphatidylethanolamines (PE) and lysophospholipids (regardless of headgroup), neither of which showed any appreciable change by multivariate analysis. Among measured metabolites involved in phospholipid biosynthesis, phosphocholine significantly increased in stool (*P* = 0.047), as shown in Fig. 4b.

The intervention significantly altered circulating phosphatidic acid membrane lipids, regardless of headgroup or whether the *sn*2 group was present (Table 4). Phosphatidylcholines largely increased (*P* < 0.0001); among the 20 PCs identified in serum, 10 significantly increased while 5 significantly decreased by univariate analysis (Supplementary Table 4). When we compared fecal and circulating PCs, all individual PCs that reached significance in stool also did so in serum; however, a consistent pattern in direction was not observed. For example, 1-palmitoyl-2-docosahexaenoyl-GPC (16:0/22:6) increased in both analyses, 1-palmitoyl-2-arachidonoyl-GPC (16:0/20:4n6) decreased in both analyses, while 1-stearoyl-2-docosahexaenoyl-GPC (18:0/22:6) decreased in feces and increased in serum. Finally, changes in circulating lysophospholipids were observed during the study (*P* < 0.0001), particularly those bearing alanine (GPA, 3/4) choline (GPC, 6/9), ethanolamine (GPE, 2/6), and glycine (GPG, 2/3) headgroups.

We also evaluated fecal collagen turnover metabolomic signatures as surrogates for intestinal extracellular matrix (ECM) structural changes. Multivariate analysis of markers of fecal collagen turnover showed significant reductions with the dietary intervention (*P* = 0.02, Table 3). Significant decreases in prohydroxyproline (*P* = 0.03), hydroxyproline (*P* = 0.0005), and 5-hydroxylysine (*P* = 0.01) were noted (Fig. 4c, Supplementary Table 3). In addition, while concentrations of circulating collagen metabolites as a group were significantly altered (*P* < 0.0001, Table 4), only the reduction in serum hydroxyproline levels reached significance (*P* = 0.002, Supplementary Table 4).

## Discussion

The impact of a complete and balanced dietary intervention containing select plant fibers was clinically evaluated in dogs with chronic large bowel diarrhea. Results from the clinical companion study demonstrated that the therapeutic food rapidly alleviated disease symptoms in the cohort [24]. Here, we consider the underlying therapeutic mechanisms of the dietary intervention through microbiome and biochemical profiling. Switching to the fiber-rich food resulted in immediate compositional changes in the stool, including significant reductions in moisture content and changes in several mineral components within 24 hours, consistent with an improved stool quality. These decreases were largely sustained over the 8-week study. A previous study evaluating an analogous food compared to a control lacking the fiber bundle observed similar reductions in moisture and ash content, which were attributed to the fiber increasing fecal organic dry matter content and improving inorganic mineral bioavailability, respectively [15]. While that study included both healthy dogs and dogs with enteritis, our study measured the impact on stool quality longitudinally in an intent-to-treat population of dogs with chronic diarrhea. Remarkably, the rapid and durable improvements in stool quality appeared to correlate with stool composition in this cohort, regardless of the subject’s pet food habits before the study and lifestyles during the study, which could not be strictly controlled.

The bacterial processes of proteolysis and saccharolysis occur in the canine hindgut and depend on the availability of substrate protein and carbohydrate, respectively, as well as the catabolic capacity of the resident microflora. Fecal metabolic profiling was employed to monitor the end products of these processes throughout the duration of the 8-week dietary intervention. Observed decreases in the metabolic products of protein degradation, including ammonia, most amino acids, several dipeptides, as well as the branched SCFAs strongly support the finding that high-fiber intervention reduced bacterial proteolytic and putrefactive processes. The polyamines encompass additional products of bacterial putrefaction, whereas decarboxylation of lysine and arginine yields cadaverine and putrescine, respectively. Subsequent addition of decarboxylated S-adenosyl methionine (SAM-dc) to putrescine generates spermine and spermidine, producing 5-methylthioadenosine (MTA) as a byproduct. Almost all measured polyamine parent and catabolic products decreased upon intervention. Intriguingly, serum metabolomics revealed increases in several circulating amino acids, suggesting the intervention improved protein digestibility and absorption in the small intestine. In contrast, pathway analysis of the microbial composition identified few changes in proteolytic capacity due to the dietary intervention, other than in tryptophan metabolic potential. Taken together, these results suggest that the intervention altered the protein-derived substrate availability for colonic microbiota, resulting in a decreased catabolic signature.

Our results also suggest an increase in saccharolytic activity by the gut microflora. An overall increase in fecal monosaccharides was observed, particularly the plant-derived compounds arabinose, mannose, xylose, and ribulose/xylulose, which may be construed as markers of microbial saccharolysis of the fiber bundle [15, 28]. Among the linear SCFAs, only butyrate showed an appreciable increase. Serum butyrate levels significantly increased over the study, suggesting that bacterially produced butyrate from fermented fiber that was not utilized locally by colonocytes was absorbed into circulation. Fecal microbiome pathway analysis supports the metabolomics differences, as only butyrate-related enzymes were altered after 24 hours of intervention. Additionally, levels of mannitol/sorbitol and ribose were decreased in stool but elevated in serum. Similarly, mannose levels increased in stool while decreasing in circulation. Taken together, these results suggest the dietary intervention differentially altered absorption of plant-derived monosaccharides by improving mannitol/sorbitol and ribose absorption, while leaving excess mannose to be eliminated. Furthermore, stool pH remained constant throughout the intervention. Given that the serum metabolomics analysis also indicated increased levels of circulating monosaccharides and showed few changes in fermentative capacity resulting from alterations in the fecal microbiome, the fiber-rich food positively impacted carbohydrate absorption before nutrients arrived in the colon, rather than altering the fermentative capacity of the colonic microbiome. Therefore, the dietary intervention likely altered the composition of the substrate available for microbial fermentation.

It is generally accepted that putrefactive postbiotics resulting from undigested protein reaching colonic bacteria can have deleterious health effects in both companion pets [29] and humans [30], including those with ulcerative colitis (UC) [31]. Conversely, a shift from proteolytic to saccharolytic microfloral catabolism is associated with positive gut health outcomes [30]. A recent human study found higher fecal levels of amino acids and peptides in patients with irritable bowel syndrome (IBS) compared to healthy controls, further demonstrating the poor outcomes associated with altered dietary protein utilization on gut health [32]. Finally, fermentative butyrate nourishes colonocytes and its serum abundance was recently shown to negatively correlate with dyslipidemia in humans [33]. In our study, increases in fecal and circulating butyrate levels may suggest that the dietary intervention may have metabolic health benefits beyond its impact on alleviating symptoms of large bowel disease.

Tryptophan catabolism in the gut relies heavily on host-microbiota interactions with several downstream functional implications. In humans, approximately 90% of serotonin is produced in the GI tract by colonic enterochromaffin (EC) cells, and resident microflora play an integral role in regulating serotonin levels [34]. Reductions in serotonin levels observed following the dietary intervention could be attributed to several factors, including reductions in tryptophan precursors, microbial-dependent attenuated production by EC cells, or possibly selection of serotonin-degrading microbes, given the significant increase in 5-hydroxyindoleacetate. Pro-inflammatory cytokines can activate indoleamine 2,3-dioxygenase and the subsequent rise in kynurenine pathway metabolites, which are implicated in controlling local and systemic inflammatory responses [35]. In our study, the high fiber food appeared to have minimal effect on kynurenine pathway stimulation (Fig. 2c); therefore, any immune-related amelioration of inflammation associated with large bowel diarrhea occurred through other mechanisms.

The presence of indole pathway metabolites in canines is attributed to microbial activity, given that mammals lack tryptophanase and other enzymes that generate these compounds. Indole pathway metabolites have been shown to be potent aryl hydrocarbon receptor (AHR) ligands in mice and humans, explaining their role in regulating intestinal barrier function and immune homeostasis [36, 37]. 3-indoxyl sulfate (3-IS) arises from a combination of host and microfloral metabolism and represents a chronic renal toxin among many mammals, including dogs [38]. The reductions in 3-IS and indole we observed in our study suggest that the intervention may have provided an additional gut-renal axis improvement.

The dietary intervention we evaluated resulted in significant enrichment of three related indole pathway metabolites in stool, suggesting that the food favored gut commensals that metabolized tryptophan to the postbiotics indoleacetate, indole-3-carboxylate, and 2-oxindole-3-acetate. Secreted by microbes in response to stress and a potent AHR agonist, indoleacetate is associated with suppression of pro-inflammatory cytokines and is often considered a beneficial postbiotic [39, 40]. Likewise, circulating indolepropionate is also suppressed in both active human UC and mouse models of colitis [41]. Restored serum levels of indolepropionate serves as a biomarker of human disease remission, which is attributed to anti-inflammatory IL-10 induction. In our study, the dietary intervention increased serum levels of indolepropionate while leaving fecal levels unchanged. We surmise that excess indolepropionate produced by gut microflora was readily absorbed and circulated, leading to its accumulation in serum. It is intriguing to speculate that circulating indolepropionate may also serve as a biomarker for the alleviation of chronic diarrhea.

A recent study investigating AHR activation identified indole-3-carboxylate as a novel human AHR ligand [42]. Interestingly, indoleacrylate, which was unchanged in the feces but significantly increased in serum in our study, was a more potent agonist than indole-3-carboxylate. Indeed, indoleacrylate production by commensal gut microbes has been shown to stimulate NRF2 gene expression, activating anti-inflammatory and antioxidant immune responses that promote intestinal epithelial barrier integrity [43]. Finally, 2-oxindole-3-acetate, as well as the serotonin catabolite 5-hydroxyindoleacetate, were significantly decreased in the stool of cats with CE [44], trends that appear to be reversed upon intervention in this study. Overall, the serum-plus-fecal signature of microbially-produced indoles may reflect an improvement in chronic large bowel diarrhea, possibly through increased synthesis of AHR ligands.

Changes in fecal levels of flavonoid species likely reflect their content in the interventional food, compared to baseline pet foods, while also providing insights into the metabolic capacities of the canine gut microbiota and their potential GI benefit. These plant-derived polyphenols are poorly absorbed in the GI tract yet are believed to be widely metabolized by resident gut microflora in both humans and pets in a manner that may influence health [45]. The anti-inflammatory properties of several of these compounds are attributed to their influence on intracellular signaling pathways, such as NF-κB and mitogen-activated protein kinases (MAPKs), and their contribution to reducing colitis severity in several rodent models has been documented in vivo [46].

The fiber bundle of the intervention contained citrus and pressed cranberries rich in flavanones, primarily hesperidin [15], a 7-O-glycoside containing the disaccharide rutinose. None of the flavanone-7-O-rutinosides measured in canine stool in our study—hesperidin, narirutin, and neoponcerin—changed appreciably in abundance with the dietary intervention. Conversely, their postbiotic flavonoid components hesperitin and naringenin significantly increased over the feeding period, as well as the disaccharide rutinose. It should be noted that ponciretin, which was also significantly elevated, is a stereoisomer of isosakuranetin, the flavonoid component of neoponcerin; therefore, neoponcerin and ponciretin may represent an additional hydrolyzed pair. Both ponciretin and poncirin were shown to suppress NF-κB and TNF-α-linked inflammation and ameliorate colitis in a chemically induced mouse model, with ponciretin demonstrating superior anti-inflammatory properties [47]. The anti-inflammatory and antioxidant properties of naringenin have been established in vitro, yet clinical data are largely lacking [48]. Hesperidin is readily hydrolyzed by rhamnosidase enzymes found in several probiotic formulations [49]. The observation that the parent glucosides from plants were unchanged while their hydrolyzed components were enriched may be indicative of increased microbial rhamnosidase activity converting flavanones to more bioavailable flavonoids in canine hindguts.

Evaluation of differences in the relative abundances of fecal phytoestrogen suggested that the dietary intervention may stimulate additional microfloral metabolic conversion. Primary dietary sources of phytoestrogens in pet foods include the flavonoid daidzein, as well as the lignans matairesinol and secoisolariciresinol, sourced from soy and flaxseed, respectively. The bioactive forms of equol, enterolactone, and enterodiol arise from colonic microbial metabolism [50]. No soy plant components were included in the intervention food, although several of the dogs were presumably consuming soy-containing food before enrollment in the study, as evident by the significant fecal depletion of daidzein throughout the feeding period. Since the intervention fiber bundle in our study contained flaxseed, matairesinol and secoisolariciresinol significantly increased in feces, as expected. Interestingly, the diglucoside of secoisolariciresinol, presumably a common form of the lignan in the flaxseed component of the food, only trended towards an increase, possibly implicating microbial activity enriching the hydrolyzed conformation. The lower levels of the phytoestrogen equol we observed were likely due to reduced availability of daidzein in the pet food. Enterolactone and enterodiol levels, on the other hand, did not correlate with changes in the concentrations of their parent lignans. Despite significant availability of the lignans secoisolariciresinol and matairesinol, we did not see increases in the microbial products enterolactone and enterodiol. These results point to suppressed microbial metabolic production of lignan-sourced bioactive phytoestrogens.

Mounting evidence implicates the endocannabinoid system, including cannabinoid receptor 1 (CB_1_) and N-acyl amide agonists, as an intersection of several nodes of host-microbiota intestinal function, including inflammation, microbial metabolism, gut permeability, and gut motility. Several studies investigating obesity in murine models suggest interplay between alterations in gut microbiota, CB_1_ receptor activation and its ligands (e.g., arachidonoyl ethanolamide) promote gut permeability, which along with obesity-induced inflammation can be readily ameliorated by CB_1_ blockade [51–53].

The rapid and consistent decreases in fecal N-acyl amides we observed with this dietary intervention appear consistent with the reduction in inflammation and improved gut permeability reported in those studies. Furthermore, endocannabinoid lipid mediators inhibit GI motility through the activation of CB_1_, which was demonstrated by exogenous addition of palmitoyl ethanolamide in a murine model of intestinal inflammation [54]. Given the improved clinical stool quality results in this study [24], we suspect the fiber intervention helped normalize colonic transit time. Since endogenous N-acyl amides are synthesized on-demand from membrane lipid precursors and are rapidly degraded after release, the slowed transit time associated with the test food may subsequently temper endogenous demand for endocannabinoids to accomplish the same physiological function, and thus reduce N-acyl amide levels relative to pre-treatment.

Initiation of the dietary intervention resulted in immediate and consistent reductions in fecal sphingolipid metabolites in our study. Sphingolipid metabolism, with ceramide at the core of this pathway, can be influenced by multiple stimuli including inflammatory responses and hypoxia [55]. Both dietary and endogenous sphingolipids are metabolized in the intestine [56], and dysregulation of sphingolipid metabolism has been implicated in several human inflammatory diseases, including UC [57]. In experimental murine models of colitis, dietary sphingomyelin exacerbated mucosal damage, colonic inflammation, and intestinal epithelial cell apoptosis in a manner suggestive of ceramide activation of the lysosomal cell death moderator cathepsin D [58]. The reduction in fecal sphingolipids observed in this study may reflect a diet-induced amelioration of inflammation and a reduction in epithelial apoptosis. Given that serum sphingomyelin levels were also largely increased, the intervention may have also mediated sphingolipid availability in the gut, whereby sphingomyelin components were more readily absorbed and increased in circulation, rather than being eliminated in the stool. While few individual sphingolipids were altered in circulation, as a class they were significantly reduced. Since elevated ceramide levels are associated with inflammatory stimuli, the reduced fecal and serum ceramide signature we observed is consistent with a modulated inflammatory state.

Alterations to both the fecal and serum PUFA profile provide additional evidence for the intervention’s alleviation of inflammation in our study. Typically, n3 and n6 PUFAs are associated with anti-inflammatory and pro-inflammatory properties, respectively. These findings are attributed to the physiology of effector lipids metabolized from either EPA (n3) or arachidonate (n6), as well as the local tissue inflammatory environment, including the types of cells present [59]. Overall, the fecal and serum n3 and n6 profiles of dogs consuming the high-fiber intervention reflected increases in the n3 PUFAs and decreases in n6 PUFAs. Since essential PUFAs are derived from food, changes from baseline in fecal PUFA levels likely reflect their relative abundances in the dietary intervention compared to pet foods the dogs were eating prior to the initiation of the intervention, while serum PUFA levels support their absorption. Indeed, the test food contains fish oil, a rich source of PUFAs, particularly n3 [15]. Additionally, the circulating eicosanoids, which are the bioactive, effector lipids metabolized by immune cell cyclooxygenase and lipoxygenase activity, largely reflected the abundances of their n3 and n6 precursors. Taken together, these profiles suggest the intervention altered the n3/n6 ratio and downstream effector abundances in a manner consistent with a reduction in pro-inflammatory precursors.

In addition to the flavonoids described above, increased abundances of additional classes of molecules found in the intervention food likely imparted nutritional benefit towards the relief of chronic large bowel diarrhea in our study. Components of vitamin E are considered potent antioxidants and radical scavengers with anti-inflammatory properties, particularly the CEHC derived from cytochrome P450-directed metabolism [60]. Systemic absorption and/or availability of these compounds was differentially impacted by the intervention, as we observed increases in circulating alpha-tocopherol and several alpha-CEHC metabolites, while gamma-tocopherol/beta-tocopherol was reduced in both stool and serum. The intervention largely increased terpenoid phytosterols, including beta-sitosterol, stigmasterol, stigmastadienone, and ergosterol. It is well documented that phytosterols can lower serum cholesterol in humans, which is attributed to their inhibition of intestinal cholesterol uptake, and potentially through regulation of cholesterol metabolism [61]. Dietary phytosterol was also shown to improve intestinal inflammation in an experimental colitis model among mice fed low-fat foods [62]. Finally, the terpenoid oleanolate has been shown to alleviate *Salmonella typhimurium*-induced diarrhea and enteritis in a mouse model through reduced NF-kB and MAPK-mediated intestinal inflammation [63]. Elevated fecal oleanolate upon intervention may also signal alleviation of chronic diarrhea in the study subjects.

While inflammatory enteropathy in the dogs in the trial was not confirmed by endoscopic biopsy, previous study has demonstrated that the underlying cause of the majority of cases of chronic diarrhea in dogs is chronic inflammatory enteropathy, including colitis and other forms of inflammatory bowel disease (IBD) [1]. IBD is associated with perturbations in host immune and microbial molecular interactions at the colonic epithelial barrier [64]. PC, ECM, and to a lesser extent sphingomyelin, are integral components of the colonic mucosal lining, providing the hydrophobic barrier that separates epithelial cells from the resident microbiota. Alterations to the phospholipid content of the colonic mucosa are implicated in the pathophysiology of UC in humans. Although the exact mechanism is unknown, patients with UC present with lower PC levels, which may be due to decreased secretion, reduced adhesion, and/or increased (host or microbe) lipase activity [65, 66]. Indeed, reintroduction of phosphatidylcholine (lecithin) may be clinically therapeutic [67]. In our study, the levels of most PC species were reduced in stool while they tended to increase in circulation. These results suggest the fiber intervention addressed PC depletion associated with inflammation, either by improved absorption in the upper GI tract, increased mucosal adhesion, and/or decreased colonic phospholipid turnover, resulting in additional systemic PC availability for the host, rather than eliminated in the feces. The latter function is further corroborated by the observed rapid increases in fecal phosphocholine, a fundamental constituent necessary for both PC and sphingomyelin biosynthesis, for which colonic depletion is associated with UC [68]. Thus, the significant and sustained increase in fecal phosphocholine with concomitant increases in circulating PCs may reflect an improvement in inflammatory enteropathy and chronic diarrhea.

Collagen fibrils constitute a significant proportion of the ECM and GI connective tissue associated with the colonic mucosa. Postranslational hydroxylation of lysine and proline residues, as well as glycosylation of the former, yields metabolic markers that reflect collagen turnover. The matrix metalloproteases (MMPs) comprise zinc-dependent endopeptidases that sculpt the ECM, including collagen. Upregulation of several secreted MMPs has been reported in the colonic mucosa of dogs with colitis, including MMP-1, MMP-3, and MMP-13 [69], as well as MMP-2 and MMP-9 [70]. Increased expression of the gelatinases MMP-2 and MMP-9 are associated with active human IBD, while their double knockout in murine models of UC is protective [71]. Collectively, these studies imply that elevated colonic mucosal collagen turnover is a consequence of colitis. Here, significant reductions from baseline in collagen markers prohydroxyproline, hydroxyproline, and 5-hydroxylysine were observed in feces, strongly suggesting that the fiber intervention modulated this presumed molecular signature of colitis pathophysiology. Moreover, human patients with IBD present with elevated serum C-terminal telopeptide, indicative of collagen fibril breakdown and typically interpreted as increased bone turnover [72]. In our study, serum hydroxyproline and collagen metabolites overall were reduced upon fiber intervention, which may be construed as suppressed collagen turnover. Further study is necessary to determine whether collagen turnover is systemic in dogs with IBD, and in turn whether the intervention ameliorated systemic collagen turnover.

A major limitation to this study was lack of a control arm; therefore, changes in microbiome composition, metabolic potential, and metabolite levels were discerned from comparisons to baseline. Additionally, not all biochemicals were detected in every patient, necessitating imputation to the observed minimum for missing metabolomic measurements. We, therefore, interpreted the data in the context of pathway changes, rather than individual biomarkers, to account for metabolites that were not uniformly detected. Controlled studies comparing the test food to a control food in healthy and diseased canines are described elsewhere [15]; however, this study was unique in its intent-to-treat objectives. Given the uncontrolled variables of the intent-to-treat population, including (but not limited to) baseline food diversity, breeds, care, and other environmental considerations, the molecular and microbiome baseline characteristics cannot be construed as a uniform disease phenotype. Another limitation is that the etiology of the dogs’ chronic diarrhea was not confirmed prior to enrollment. Although veterinarians in the trial thoroughly evaluated the dogs based on their medical history, clinical signs, physical examinations, and laboratory analyses, endoscopic biopsies were not conducted. Because of the high rates of response to dietary interventions in dogs with chronic diarrhea, dietary changes are typically recommended in dogs with chronic diarrhea before they undergo invasive diagnostic procedures. Consequently, conducting invasive diagnostic procedures may have subjected patients to unnecessary discomfort and would have violated the sponsor’s current animal welfare standards for clinical studies. However, the fact that we saw significant alterations in pathways associated with the pathophysiology of the primary causes of chronic large bowel diarrhea indicates the potential for a fiber-based intervention to drive the GI and systemic health of these canines.

## Conclusions

Feeding of a high-fiber dietary intervention to dogs with chronic diarrhea resulted in numerous underlying changes to the dogs’ microbiomes and metabolomes. While subtle compositional changes to the canine fecal microflora accompanied the intervention, the treatment resulted in extensively different fecal and circulating metabolic signatures relative to baseline. Several lines of evidence in the metabolomic data supported a shift from putrefactive to saccharolytic fermentation by colonic bacteria, likely by altered amino acid and carbohydrate availability. Combined metagenomic and metabolomic pathway mapping of fecal tryptophan metabolism indicated the food favored the production of largely beneficial indole-containing catabolites, several of which also increased in circulation. Additional flavonoid, lignan, and other plant-derived microbial postbiotics with favorable profiles were enriched in feces. The intervention significantly affected lipid metabolites, particularly sphingolipids and PCs, in a manner consistent with an improvement in the pathophysiology of inflammatory enteropathy and large bowel diarrhea, as well as endocannabinoids and PUFAs that may reflect normalization in colonic transit time and inflammation, respectively. Signatures of an improved colonic mucosal barrier were also evident from phospholipid and collagen metabolites. Overall, the high-fiber intervention appeared to reduce the signs of chronic diarrhea and positively influence gut microbial and inflammatory metabolism by multiple mechanisms.

## Methods

This was a prospective, single-arm, 8-week clinical study of adult dogs with chronic diarrhea treated in 11 private practices in the United States. The study was conducted between March 2017 and March 2018. The study protocol was reviewed and approved by the Institutional Animal Care and Use Committee, Hill’s Pet Nutrition, Inc., in Topeka, Kansas (permit number, 719.0.0). Procedures were designed to avoid or minimize pain, discomfort, or distress, and dogs were monitored for any signs of disease. The dog’s health always took precedence over continuation in the study in the case of an adverse event. Owners signed an informed consent form before their dog was enrolled and agreed to comply with the instructions given by the veterinarian and listed in the protocol.

### Study population

Dogs were eligible for inclusion if they were aged 1 to 10 years, had experienced chronic diarrhea, and were currently exhibiting unresolved diarrhea (primarily large bowel diarrhea) with frequent emission of feces, liquid or loose stool consistency, straining while defecating (dyschezia), frequent attempts to evacuate bowels (tenesmus), displays of abdominal discomfort, blood in stool (hematochezia), mucus in stool, vomiting, or loss of appetite. To be eligible, dogs’ current episode of diarrhea must have lasted at least 2 weeks. Veterinarians’ assessments were based on the dogs’ medical history, clinical signs, and results of physical examinations and laboratory analyses.

### Dietary intervention

All enrolled dogs were assigned to the test food (Hill’s Prescription Diet Gastrointestinal Biome), a complete and balanced dry therapeutic formulation that included whole grains and the following sources of fiber: ground pecan shells, cellulose, flaxseed, dried beet pulp, dried citrus pulp, pressed cranberries, dried pumpkin, and psyllium seed husks. The formulation also contained chicken, barley, ginger root, fish oil, taurine, vitamins, and minerals. Pet owners were instructed to avoid offering treats for the duration of the study, to avoid giving the study food to other pets, and to ensure that water is consistently available to the pet. Investigators and pet owners were blinded to the identity of the Sponsor and to the assigned study food.

### Sample collection

Baseline fecal and stool samples were collected on Day 1, after which the study participants were fed the high-fiber intervention food by their owners. Stool specimens were collected on Days 1, 2, 3, 14, 28, and 56. Serum samples were obtained on Days 1, 2, 3, 28, and 56.

### Fecal proximate analyses and mineral composition of ash

Whole feces were collected after defecation, homogenized thoroughly by hand until visually uniform, snap-frozen in liquid nitrogen, and stored at − 80 °C until further processing. Proximate, vitamin, amino acid, and fatty acid analyses were conducted using certified official compendial methods in ISO-accredited commercial laboratories. Moisture of fecal samples was evaluated by spreading feces in an aluminum pan and drying for approximately 3 hours. Ash values were assessed by weighing a portion of the fecal sample in a small ceramic crucible and heating to 600 °C for approximately 2 hours.

### Metabolomic profiling

Fecal and serum metabolomics profiling were performed by Metabolon, Inc. (Morrisville, NC), as previously described [73]. Briefly, samples were extracted in methanol and split into equal parts for analysis on four methods employing exact-mass Q-Exactive mass spectrometers (MS): two reverse phase (RP)/ultra-high performance liquid chromatography-tandem-MS (UPLC-MS/MS) in positive ion mode, one RP/UPLC-MS/MS in negative ion mode, and one polar (HILIC)/UPLC-MS/MS in negative ion mode. Proprietary software was used to match ions to an in-house library of authentic and in silico standards for metabolite identification and subsequent peak area integration. Automatic and manual QC were performed on the resulting datasets prior to statistical analysis. Compounds with missing values (i.e., were measured in some but not all dogs) were assumed to be below the limit of detection, and thus were imputed with the observed minimum for that biochemical. Metabolite assignments that lack authentic standards, yet still demonstrate high confidence in chemical identification are marked with an asterisk (*). Metabolites with identical masses (isobar) that could not be distinguished by chromatography and thus confidently assigned were named accordingly (e.g., ribulose/xylulose).

### Evaluation of the microbiome: DNA extraction, 16S rDNA amplicon sequencing, and processing

Total DNA was extracted from frozen feces samples using the PowerFecal DNA isolation kit (MOBIO, Carlsbad, CA), following the instructions of the manufacturer. However, a sonication step was introduced before vortexing the bead tubes with fecal samples horizontally for 15 minutes, as described by Jackson and colleagues [15]. PCR amplification and 50 Amplicon sequencing were performed, using the primer pairs 341F and 806R spanning the V3-V4 hypervariable regions of the 16S rRNA gene along with Illumina adapters, and the Illumina 16S metagenomic sequencing library preparation protocol (15,044,223 Rev. A), respectively. The sequences were de-multiplexed based on the dual index sequences, using the Miseq built-in metagenomics workflow to develop FASTQ files. Data are available upon request.

### Statistical analysis

Calculation of the sample size for enrollment has been previously described [24].

Fecal moisture, pH, fatty acid, ash and mineral concentration data were analyzed using a general linear model with Day as the fixed effect. To account for the correlation between the repeated measurements on each dog over time, a preliminary analysis was conducted in which compound symmetry, compound symmetry heterogeneous, first-order ante-dependence, and unstructured covariance structures were fit to the data. The final model was selected using the AICC and BIC fit statistics. The mean scores from all post-Day 1 time points were compared to Day 1 mean scores using a two-sided t-test with the DIFF=CONTROL option in the least-squares means statement. All analyses were performed using PROC MIXED or PROC GLIMMIX in SAS®, version 9.2 or version 9.4. All results were considered statistically significant at the 0.10 significance level.

#### Microbiome analysis

The alpha-diversity indices were analyzed using Friedman’s test. The 16S copy number-corrected OTU counts and PICRUSt-predicted functional data were filtered by prevalence. They had to pass 80% prevalence in at least one of the time points to be considered for further statistical analysis. The counts of individual OTUs and predicted KO functions were analyzed by negative binomial mixed models [74]. Permutational multivariate analysis of variance (PERMANOVA) based on Manhattan distance was used to compare relative abundances of microbial compositions and functional KO compositions of selected individual pathways between time points [75]. The selected pathways included arginine, benzoate, butyrate, phenylalanine, propionate, tryptophan, and tyrosine. *P*-values were FDR adjusted according to the Benjamini and Hochberg procedure [76]. All the statistical microbiome analyses were performed in R-3.3.3 [77].

#### Metabolomics analysis

Scaled metabolomic data collected at Days 1, 2, 3, 14 (fecal only), 28, and 56 were organized into 39 (fecal) or 21 (serum) functional groups. A MANOVA with Day as the fixed effect in the model was performed for each group of metabolites to test for significant linear, quadratic, or cubic trends over time using orthogonal polynomial contrasts. Because the days were unequally spaced, coefficients for the orthogonal polynomial contrasts were calculated using the MANOVA option in PROC GLM.

In addition, each of the individual chemicals within the groups were analyzed using a univariate analysis of variance with Day as the fixed effect in the model. To account for the correlation between the repeated measurements made at each time point, compound symmetry, compound symmetry heterogeneous, spatial power, first-order ante-dependence, and unstructured covariance structures were fit to the data, and the most appropriate covariance structure for each metabolite was selected using the AICC fit statistic. Orthogonal polynomial contrasts were used to test for linear, quadratic, and cubic trends over days for each individual metabolite.

#### Combining Functional observations with metabolite changes through pathway mapping

To evaluate consistency and attempt to develop a mechanistic perspective for the observed changes in microbiome composition and metabolite abundance in fecal samples, the differences in functional composition inferred from microbiome composition were projected onto relevant modified KEGG [25] pathways.

Microbial composition estimates at genus level for each sample produced through Mothur [78] using MiSeq SOP [79] were translated into functional compositions using PICRUSt [80] with Greengenes V13.5 as a reference database. The resulting (inferred) abundance estimates for each KEGG ortholog (KO) were individually evaluated for statistically significant differences between initial and final state of the study and combined with independently evaluated metabolite changes for the same sample groups. Effect sizes were discretized into ‘increased’, ‘decreased’ and ‘unchanged’ and projected onto relevant KEGG pathways.

Discretized observations were mapped onto individual pathways via KOs and chemical identifiers, respectively, and colored accordingly. Ambiguous enzymatic entities were removed and replaced with simplified representations (direct arrows) where required to maintain as complete a representation of the original KEGG pathway as necessary. Pathway editing and rendering was performed using Cytoscape V 3.8.0 [81].

## Supplementary Information

**Additional file 1.**

**Additional file 2.**

**Additional file 3.**

**Additional file 4.**

## Data Availability

The datasets used and/or analyzed during the current study are available from the corresponding author upon reasonable request.

## Abbreviations

3-IS: 3-indoxyl sulfate
12-HETE: 12-hydroxyeicosatetraenoic acid
12-HHTrE: 12-hydroxyheptadecatrienoic acid
ANOVA: analysis of variation
CB_1_: cannabinoid receptor 1
CE: chronic enteropathy
CEHC: carboxyethyl hydroxychroman
ECM: extracellular matrix
EPA: eicosapentaenoic acid
GI: gastrointestinal
HCER: hexosylceramide
IBD: inflammatory bowel disease
IBS: inflammatory bowel syndrome
KEGG: Kyoto Encyclopedia of Genes and Genomes
LCER: lactosylceramide
MANOVA: multivariate analysis of variation
MMP: matrix metalloprotease
MTA: 5′-methylthioadenosine
PICRUSt: phylogenetic investigation of communities by reconstruction of unobserved states
PC: phosphatidylcholine
PE: phosphatidylethanolamine
SCFA: short-chain fatty acid
UC: ulcerative colitis
