# Supplementary material for: Microbiome function underpins the efficacy of a fiber-supplemented dietary intervention in dogs with chronic large bowel diarrhea
Source: BMC Vet Res. 2022 Jun 24;18:245. doi: 10.1186/s12917-022-03315-3 (PMC9233311; doi:10.1186/s12917-022-03315-3)
Supplement: Supplementary file 1 — Additional file 1. [file 12917_2022_3315_MOESM1_ESM.docx]

**Supplementary Table 1**. Fecal composition from canines with colitis consuming intervention diet

| Fecal  Compositional Analysis | Day 1 | | Day 3 | | Difference From Day 1 to Day 3 | Day 56 | | Difference  From Day 1 to Day 56 |
| --- | --- | --- | --- | --- | --- | --- | --- | --- |
|  | n | Mean (SE) | n | Mean  (SE) | Mean (SE) | n | Mean  (SE) | Mean (SE) |
| Moisture (%) | 26 | 70.08  (1.20) | 30 | 68.13  (0.78) | -1.96 (1.15)* | 22 | 68.63  (0.65) | -1.45 (1.14) |
| pH | 27 | 5.91  (0.26) | 30 | 6.05  (0.06) | 0.15 (0.27) | 22 | 5.89  (0.04) | -0.01 (0.26) |
| Ash (%) | 26 | 6.57  (0.63) | 30 | 4.54  (0.15) | -2.03 (0.63)** | 22 | 4.76  (0.19) | -1.81 (0.64)** |
| Ammonium  (mmol/g) | 27 | 0.040  (0.004) | 30 | 0.028  (0.002) | -0.0113  (0.004)** | 22 | 0.024  (0.001) | -0.0155  (0.004)*** |
| Calcium (ppm) | 26 | 57,071 (4772) | 29 | 41505  (983) | -15567  (4686)*** | 22 | 42951  (837) | -14120  (4692)** |
| Copper (ppm) | 26 | 75.7  (4.7) | 29 | 85.3  (6.3) | 9.6 (7.5) | 22 | 67.3  (4.0) | -8.4 (6.2) |
| Iron (ppm) | 26 | 957  (92) | 29 | 1214  (93) | 257 (122) | 22 | 1419  (140) | 461 (167) |
| Magnesium  (ppm) | 26 | 4167  (296) | 29 | 3537  (402) | -631 (450) | 22 | 3300  (106) | -868 (295)** |
| Manganese  (ppm) | 26 | 203  (25) | 29 | 166 (3) | -37 (25) | 22 | 190 (8) | -12 (25) |
| Potassium  (ppm) | 26 | 3056  (448) | 29 | 3216  (301) | 160 (403) | 22 | 2304  (161) | -752 (476) |
| Sodium (ppm) | 26 | 3641  (741) | 29 | 2735  (254) | -907 (0.089) | 22 | 2900  (270) | -741 (674) |
| Phosphorus  (ppm) | 26 | 28751  (2486) | 29 | 19713  (428) | -9038  (2463)*** | 22 | 20932  (571) | -7819 (2475)** |
| Zinc (ppm) | 26 | 1231  (91) | 29 | 1307  (79) | 76 (104) | 56 | 22  (1200) | -31 (92) |

*P < 0.05; **P < 0.01; ***P < 0.001.
