# Supplementary material for: Microbiome function underpins the efficacy of a fiber-supplemented dietary intervention in dogs with chronic large bowel diarrhea
Source: BMC Vet Res. 2022 Jun 24;18:245. doi: 10.1186/s12917-022-03315-3 (PMC9233311; doi:10.1186/s12917-022-03315-3)
Supplement: Supplementary file 2 — Additional file 2. [file 12917_2022_3315_MOESM2_ESM.docx]

**Table S2.** Significant day effects in the selected PICRUSt-predicted functional pathways.

| Study Day | KEGG orthology (KO) group | Value* | *P* value | FDR corrected *P* value |
| --- | --- | --- | --- | --- |
| DayT-02 | K00023 | -1.351 | 0.0026 | 0.0109 |
| DayT-02 | K00169 | -0.717 | 0.0104 | 0.0386 |
| DayT-02 | K00170 | -0.734 | 0.0094 | 0.0359 |
| DayT-02 | K00172 | -0.717 | 0.0105 | 0.0386 |
| DayT-02 | K01426 | -0.631 | 0.0070 | 0.0273 |
| DayT-02 | K01617 | -1.164 | 0.0082 | 0.0314 |
| DayT-02 | K01907 | -1.247 | 0.0026 | 0.0109 |
| DayT-02 | K01908 | -1.144 | 0.0008 | 0.0034 |
| DayT-02 | K01963 | 0.126 | 0.0078 | 0.0301 |
| DayT-02 | K04072 | 0.151 | 0.0044 | 0.0175 |
| DayT-02 | K07516 | -1.302 | 0.0051 | 0.0204 |
| DayT-03 | K00023 | -1.415 | 0.0017 | 0.0071 |
| DayT-03 | K00451 | -1.017 | 0.0090 | 0.0343 |
| DayT-03 | K01426 | -0.709 | 0.0025 | 0.0107 |
| DayT-03 | K01580 | -0.728 | 0.0116 | 0.0420 |
| DayT-03 | K01692 | -0.833 | 0.0067 | 0.0264 |
| DayT-03 | K01907 | -1.218 | 0.0033 | 0.0134 |
| DayT-03 | K01963 | 0.122 | 0.0101 | 0.0377 |
| DayT-03 | K03781 | -0.667 | 0.0104 | 0.0386 |
| DayT-03 | K07516 | -1.219 | 0.0086 | 0.0331 |
| DayT-14 | K00135 | 0.885 | 0.0013 | 0.0057 |
| DayT-14 | K00140 | 0.851 | 0.0063 | 0.0245 |
| DayT-14 | K00242 | 0.951 | 0.0036 | 0.0147 |
| DayT-14 | K00285 | 1.001 | 0.0033 | 0.0136 |
| DayT-14 | K00446 | 1.242 | 0.0107 | 0.0393 |
| DayT-14 | K00448 | 1.138 | 0.0050 | 0.0200 |
| DayT-14 | K00449 | 1.121 | 0.0036 | 0.0148 |
| DayT-14 | K00457 | 1.313 | 0.0002 | 0.0009 |
| DayT-14 | K01031 | 1.370 | 0.0003 | 0.0012 |
| DayT-14 | K01032 | 1.373 | 0.0003 | 0.0012 |
| DayT-14 | K01055 | 1.600 | 0.0000 | 0.0001 |
| DayT-14 | K01457 | 1.061 | 0.0042 | 0.0170 |
| DayT-14 | K01682 | 0.915 | 0.0125 | 0.0451 |
| DayT-14 | K01821 | 0.742 | 0.0124 | 0.0449 |
| DayT-14 | K01826 | 1.087 | 0.0140 | 0.0499 |
| DayT-14 | K01941 | 1.256 | 0.0006 | 0.0028 |
| DayT-14 | K02609 | 1.065 | 0.0075 | 0.0291 |
| DayT-14 | K02610 | 1.151 | 0.0040 | 0.0163 |
| DayT-14 | K02611 | 1.088 | 0.0070 | 0.0271 |
| DayT-14 | K02612 | 1.089 | 0.0070 | 0.0271 |
| DayT-14 | K02613 | 1.091 | 0.0028 | 0.0116 |
| DayT-14 | K03366 | 1.109 | 0.0044 | 0.0175 |
| DayT-14 | K03381 | 1.179 | 0.0019 | 0.0079 |
| DayT-14 | K03464 | 1.432 | 0.0002 | 0.0009 |
| DayT-14 | K04073 | 1.091 | 0.0121 | 0.0440 |
| DayT-14 | K05597 | 1.179 | 0.0078 | 0.0301 |
| DayT-14 | K05783 | 0.938 | 0.0098 | 0.0372 |
| DayT-14 | K14682 | 1.281 | 0.0006 | 0.0028 |
| DayT-14 | K14727 | 1.153 | 0.0115 | 0.0420 |

| DayT-28 | K01555 | -1.086 | 0.0052 | 0.0206 |
| --- | --- | --- | --- | --- |
| DayT-28 | K01580 | -0.729 | 0.0114 | 0.0416 |
| DayT-28 | K01659 | -1.385 | 0.0003 | 0.0013 |
| Study Day | KEGG orthology (KO) group | Value* | *P* value | FDR corrected *P* value |
| DayT-28 | K01847 | -0.491 | 0.0103 | 0.0384 |
| DayT-28 | K01963 | 0.173 | 0.0003 | 0.0013 |
| DayT-28 | K02160 | 0.176 | 0.0026 | 0.0109 |
| DayT-28 | K04072 | 0.147 | 0.0056 | 0.0222 |
| DayT-28 | K08325 | -0.818 | 0.0102 | 0.0380 |
| DayT-28 | K15036 | -2.205 | 0.0000 | 0.0000 |
| DayT-56 | K00276 | -1.698 | 0.0005 | 0.0022 |
| DayT-56 | K00285 | 0.985 | 0.0038 | 0.0156 |
| DayT-56 | K00448 | 1.196 | 0.0033 | 0.0134 |
| DayT-56 | K00449 | 1.134 | 0.0033 | 0.0136 |
| DayT-56 | K00451 | -1.343 | 0.0008 | 0.0035 |
| DayT-56 | K01031 | 1.434 | 0.0002 | 0.0007 |
| DayT-56 | K01032 | 1.437 | 0.0001 | 0.0007 |
| DayT-56 | K01457 | 0.956 | 0.0097 | 0.0368 |
| DayT-56 | K01653 | -0.091 | 0.0015 | 0.0065 |
| DayT-56 | K01721 | -1.616 | 0.0023 | 0.0096 |
| DayT-56 | K01847 | -0.527 | 0.0060 | 0.0235 |
| DayT-56 | K01857 | -1.118 | 0.0068 | 0.0265 |
| DayT-56 | K01941 | 1.080 | 0.0031 | 0.0129 |
| DayT-56 | K01963 | 0.162 | 0.0007 | 0.0029 |
| DayT-56 | K02160 | 0.180 | 0.0021 | 0.0088 |
| DayT-56 | K02554 | -1.605 | 0.0031 | 0.0128 |
| DayT-56 | K02610 | -1.042 | 0.0135 | 0.0486 |
| DayT-56 | K02611 | -1.092 | 0.0106 | 0.0389 |
| DayT-56 | K02612 | -1.091 | 0.0106 | 0.0389 |
| DayT-56 | K03366 | 1.004 | 0.0096 | 0.0366 |
| DayT-56 | K04072 | 0.176 | 0.0010 | 0.0042 |
| Study Day | KEGG orthology (KO) group | Value* | *P* value | FDR corrected *P* value |
| DayT-56 | K04103 | 0.977 | 0.0127 | 0.0456 |
| DayT-56 | K05712 | -1.601 | 0.0025 | 0.0103 |
| DayT-56 | K08318 | 1.601 | 0.0009 | 0.0038 |
| DayT-56 | K08324 | 1.327 | 0.0032 | 0.0134 |
| DayT-56 | K09788 | -1.058 | 0.0050 | 0.0200 |
| DayT-56 | K11258 | 1.033 | 0.0104 | 0.0386 |
| DayT-56 | K14682 | 1.285 | 0.0006 | 0.0027 |
| DayT-56 | K14727 | 1.957 | 0.0000 | 0.0001 |
| DayT-56 | K15036 | -1.374 | 0.0016 | 0.0070 |

* Values are the estimated log ratios of relative pathway abundances between the respective Day and Day1
