# Supplementary material for: Microbiome function underpins the efficacy of a fiber-supplemented dietary intervention in dogs with chronic large bowel diarrhea
Source: BMC Vet Res. 2022 Jun 24;18:245. doi: 10.1186/s12917-022-03315-3 (PMC9233311; doi:10.1186/s12917-022-03315-3)
Supplement: Supplementary file 3 — Additional file 3. [file 12917_2022_3315_MOESM3_ESM.pdf]

Supplementary Table 3 - Fecal Metabolomics

		Day linear	Day quad	Day cubic	Change
Alkaloids	**Manova**	0.0071	0.0120	0.7449	
piperine		0.9730	0.1532	0.5021	
piperidine		0.6668	0.6749	0.0987	
nicotianamine		0.0105	0.0773	0.3920	Decreased
stachydrine		0.4882	0.3468	0.7110	
homostachydrine		0.1290	0.2014	0.3203	
pyrraline		0.2696	0.9283	0.7915	
ergothioneine		0.0012	0.0041	0.0625	Decreased
deoxymugineic acid		0.0008	0.0009	0.0285	Increased
1-methyl-beta-carboline-3-carboxylic acid		0.5663	0.0111	0.1745	Decreased
DIMBOA		<.0001	<.0001	0.0337	Increased
3-hydroxystachydrine		0.1574	0.5298	0.1207	
solanidine		0.0954	0.0897	0.0953	decreased
dipicolinate		0.7726	0.1153	0.1303	
Amino Acids	**Manova**	0.0008	0.4713	0.8579	Decreased
glycine		<.0001	0.0027	0.0210	Decreased
serine		0.0001	0.0116	0.0180	Decreased
threonine		<.0001	0.0010	0.0245	Decreased
alanine		0.0038	0.0007	0.0704	Decreased
aspartate		0.0034	0.0179	0.1778	Decreased
asparagine		0.1547	0.4182	0.0133	Decreased
glutamate		0.0093	0.0120	0.1112	Decreased
glutamine		0.0003	0.0607	0.4242	Decreased
histidine		0.2074	0.0712	0.7834	decreased
lysine		0.0010	0.0030	0.0477	Decreased
phenylalanine		<.0001	0.0191	0.0268	Decreased
tyrosine		0.0005	0.0049	0.0556	Decreased
tryptophan		0.1059	0.2509	0.0466	Decreased
leucine		<.0001	0.0069	0.0198	Decreased
isoleucine		0.0003	0.0895	0.1121	Decreased
valine		<.0001	0.0039	0.0053	Decreased
methionine		<.0001	0.0082	0.0778	Decreased
cysteine		0.3812	0.1340	0.8174	
taurine		0.0421	0.1712	0.5348	Decreased
arginine		0.1907	0.0656	0.6628	decreased
proline		<.0001	0.0009	0.0420	Decreased
Benzoate Metabolism	**Manova**	0.0754	0.0302	0.2884	
4-hydroxybenzoate		0.0419	0.3041	0.2702	Increased
2,4,6-trihydroxybenzoate		0.0026	<.0001	0.0001	Increased
2-(4-hydroxyphenyl)propionate		0.1069	0.1763	0.2266	
3-(4-hydroxyphenyl)propionate		0.1622	0.0109	0.4250	Increased
3-(3-hydroxyphenyl)propionate		0.8824	0.9904	0.2138	
3-phenylpropionate (hydrocinnamate)		0.7271	0.3811	0.0406	

Supplementary Table 3 - Fecal Metabolomics

		Day linear	Day quad	Day cubic	Change
Carbohydrate Metabolism	**Manova**	0.0017	0.7670	0.8289	
arabinose		0.0038	0.3739	0.0097	Increased
arabitol/xylitol		0.1657	0.4206	0.1961	
arabonate/xylonate		0.3759	0.7747	0.3992	
erythose		0.6209	0.0099	0.0531	Decreased
erythritol		0.4466	0.7808	0.1952	
erythronate*		0.8429	0.1254	0.5754	
fructose		0.8712	0.1370	0.7152	
fucose		0.6582	0.1329	0.3902	
galactitol (dulcitol)		0.6992	0.0157	0.4352	Decreased
galactonate		0.7972	0.5232	0.6439	
glucose		0.3042	0.5083	0.6538	
glucuronate		0.8681	0.0212	0.1459	Decreased
glycerate		0.5908	0.2503	0.9223	
lactate		0.9138	0.0861	0.9369	
maltose		0.2731	0.5660	0.2021	
mannitol/sorbitol		0.0658	0.0581	0.6497	decreased
mannonate*		0.7593	0.5133	0.6376	
mannose		0.0283	0.8655	0.8153	Increased
pyruvate		0.4979	0.3000	0.6295	
ribitol		0.3824	0.5023	0.7363	
ribonate		0.3230	0.2495	0.7687	
ribose		0.0141	0.0134	0.3262	Decreased
ribulonate/xylulonate*		0.3079	0.8936	0.6781	
ribulose/xylulose		0.0035	0.3115	0.0695	Increased
xylose		0.0060	0.3982	0.5093	Increased
Collagen Metabolism	**Manova**	0.0192	0.1197	0.5714	Decreased
prolylhydroxyproline		0.0340	0.0560	0.0740	Decreased
5-hydroxylysine		0.0937	0.0109	0.1303	Decreased
5-(galactosylhydroxy)-L-lysine		0.0849	0.0513	0.1652	
hydroxyproline		<.0001	0.0005	0.0579	Decreased
Dipeptides	**Manova**	0.0011	0.5644	0.9675	Decreased
alanylleucine		0.4931	0.1496	0.2749	
glutaminylleucine		0.1614	0.6135	0.6643	
glycylisoleucine		0.0393	0.8996	0.2095	Decreased
glycylleucine		0.0524	0.3206	0.3643	
glycylvaline		0.0050	0.2854	0.3232	Decreased
isoleucylglycine		0.0673	0.1477	0.1043	
leucylalanine		0.5957	0.1127	0.2268	
leucylglycine		0.4211	0.4177	0.4559	
lysylleucine		0.2795	0.0299	0.2175	Decreased
phenylalanylalanine		0.1692	0.0448	0.3015	Decreased
phenylalanylglycine		0.8129	0.3736	0.3881	
prolylglycine		0.0371	0.0203	0.1019	Decreased
threonylphenylalanine		0.4537	0.2721	0.2691	
tryptophylglycine		0.0511	0.0590	0.1268	
tyrosylglycine		0.5833	0.0333	0.5984	Decreased
valylglutamine		0.8677	0.6597	0.2160	
valylglycine		0.3241	0.2548	0.0868	
valylleucine		0.4847	0.3835	0.9188	
leucylglutamine*		0.8293	0.1723	0.7050	

Supplementary Table 3 - Fecal Metabolomics

		Day linear	Day quad	Day cubic	Change
Endocannabinoids	**Manova**	0.0244	0.1927	0.8042	**Decreased**
oleoyl ethanolamide		0.0011	0.0032	0.0341	Decreased
palmitoyl ethanolamide		0.0001	0.0001	0.0008	Decreased
stearoyl ethanolamide		<.0001	<.0001	0.0004	Decreased
dihomo-linolenoyl ethanolamide		<.0001	<.0001	<.0001	Decreased
arachidonoyl ethanolamide		<.0001	<.0001	0.0001	Decreased
linoleoyl ethanolamide		0.0180	0.0280	0.4450	Decreased
linolenoyl ethanolamide		0.0137	0.0108	0.1599	Decreased
arachidoyl ethanolamide (20:0)*		<.0001	<.0001	0.0028	Decreased
behenoyl ethanolamide (22:0)*		<.0001	<.0001	0.0083	Decreased
erucoyl ethanolamide (22:1)*		<.0001	<.0001	0.0104	Decreased
lignoceroyl ethanolamide (24:0)*		<.0001	<.0001	0.0155	Decreased
nervonoyl ethanolamide (24:1)*		<.0001	<.0001	0.0126	Decreased
palmitoleoyl ethanolamide*		0.0007	0.0002	0.0024	Decreased
margaroyl ethanolamide*		<.0001	0.0003	0.0015	Decreased
N-palmitoylserine		0.4218	0.1388	0.2949	
Polyunsaturated n3 Fatty Acids	**Manova**	0.0125	0.0168	0.1472	
linolenate (18:3n3 or 3n6)		0.3323	0.0008	0.0003	Increased
hexadecatrienoate (16:3n3)		0.1239	0.1866	0.8905	
stearidonate (18:4n3)		0.0023	0.0844	0.3956	Increased
eicosapentaenoate (EPA; 20:5n3)		0.0149	0.5395	0.3093	Increased
heneicosapentaenoate (21:5n3)		0.1461	0.8266	0.2045	
docosatrienoate (22:3n3)		0.0089	0.6686	0.2560	Decreased
docosapentaenoate (DPA; 22:5n3)		0.5859	0.6373	0.7609	
docosahexaenoate (DHA; 22:6n3)		0.7707	0.2259	0.8595	
Polyunsaturated n6 Fatty Acids	**Manova**	0.0159	0.6641	0.4808	Decreased
hexadecadienoate (16:2n6)		0.7641	0.7074	0.3962	
linoleate (18:2n6)		0.6413	0.6725	0.1617	
dihomolinoleate (20:2n6)		0.0408	0.1145	0.6696	Decreased
arachidonate (20:4n6)		0.0611	0.0789	0.3073	decreased
docosadienoate (22:2n6)		0.0960	0.0833	0.8990	decreased
docosatrienoate (22:3n6)*		0.0360	0.1672	0.2609	Decreased
adrenate (22:4n6)		<.0001	0.0079	0.4831	Decreased
docosapentaenoate (n6 DPA; 22:5n6)		0.0663	0.0501	0.2029	decreased
Saturated and Monounsaturated Fatty Acids	**Manova**	0.0611	0.5269	0.4188	decreased
valerate (5:0)		0.0027	0.0036	0.0698	Decreased
caproate (6:0)		0.0109	0.0312	0.1995	Decreased
caprylate (8:0)		0.7999	0.5717	0.3107	
caprate (10:0)		0.0269	0.1339	0.2938	Decreased
5-dodecenoate (12:1n7)		0.7533	0.9023	0.0837	decreased
myristoleate (14:1n5)		0.0368	0.0913	0.0056	Decreased
pentadecanoate (15:0)		0.1640	0.6877	0.1755	
palmitoleate (16:1n7)		0.4755	0.5511	0.8975	
margarate (17:0)		0.3594	0.3778	0.5040	
oleate/vaccenate (18:1)		0.5721	0.4068	0.1874	
nonadecanoate (19:0)		0.4340	0.5774	0.1187	
10-nonadecenoate (19:1n9)		0.4100	0.0851	0.9644	decreased
eicosenoate (20:1n9 or 1n11)		0.7332	0.1113	0.4161	
erucate (22:1n9)		0.7351	0.4580	0.0446	
Hemoglobin Metabolism	**Manova**	0.0590	0.7983	0.7122	decreased
heme		0.1581	0.2470	0.3321	
bilirubin		<.0001	0.0549	0.2404	Decreased
bilirubin (E,E)*		0.0018	0.1049	0.1248	Decreased
bilirubin (E,Z or Z,E)*		0.3263	0.2272	0.6678	
biliverdin		0.0001	0.0123	0.5011	Decreased
I-urobilinogen		0.0089	0.0229	0.8919	Decreased
D-urobilin		0.0696	0.0108	0.0993	Decreased
L-urobilin		0.7040	0.6674	0.1316	

Supplementary Table 3 - Fecal Metabolomics

		Day linear	Day quad	Day cubic	Change
Linolenate Metabolism	**Manova**	0.0510	0.0643	0.0253	**Increased**
linolenate (18:3n3 or 3n6)		0.3323	0.0008	0.0003	Increased
dihomo-linolenate (20:3n3 or 3n6)		0.0004	0.1551	0.5063	Decreased
1-palmitoyl-2-linolenoyl-galactosylglycerol (16:0/18:3)*		0.3107	0.5174	0.7593	
1-palmitoyl-2-linolenoyl-digalactosylglycerol (16:0/18:3)		0.3657	0.5064	0.5298	
1-linoleoyl-2-linolenoyl-galactosylglycerol (18:2/18:3)*		0.2844	0.8703	0.9652	
1-linoleoyl-2-linolenoyl-digalactosylglycerol (18:2/18:3)*		0.5934	0.8852	0.9296	
1,2-dilinolenoyl-digalactosylglycerol (18:3/18:3)		0.3305	0.5057	0.5716	
1,2-dilinolenoyl-galactosylglycerol (18:3/18:3)*		0.3073	0.6535	0.6684	
1-linolenoylglycerol (18:3)		0.5840	0.2816	0.0033	Increased
palmitoyl-linolenoyl-glycerol (16:0/18:3) [2]*		0.1499	0.8231	0.3477	
stearoyl-linolenoyl-glycerol (18:0/18:3) [2]*		0.1941	0.4922	0.0155	Increased
oleoyl-linolenoyl-glycerol (18:1/18:3) [2]*		0.4283	0.7880	0.0450	Increased
linoleoyl-linolenoyl-glycerol (18:2/18:3) [1]*		0.0665	0.9528	0.2343	
linoleoyl-linolenoyl-glycerol (18:2/18:3) [2]*		0.0855	0.6743	0.0757	
linolenoyl-linolenoyl-glycerol (18:3/18:3) [1]*		0.4151	0.4379	0.1406	
linolenoyl-linolenoyl-glycerol (18:3/18:3) [2]*		0.2125	0.4382	0.0171	Increased
Monoacylglycerols	**Manova**	0.0401	0.4209	0.3265	
1-pentadecanoylglycerol (15:0)		0.5365	0.4333	0.4616	
1-palmitoleoylglycerol (16:1)*		0.7276	0.8936	0.0750	
1-oleoylglycerol (18:1)		0.4403	0.4578	0.1264	
1-linoleoylglycerol (18:2)		0.8492	0.9522	0.1078	
1-linolenoylglycerol (18:3)		0.5840	0.2816	0.0033	Increased
1-docosahexaenoylglycerol (22:6)		0.0038	0.3552	0.3295	Increased
2-oleoylglycerol (18:1)		0.3800	0.2919	0.0759	
2-linoleoylglycerol (18:2)		0.3254	0.1291	0.1699	
Phenolic Compounds	**Manova**	0.0756	0.3573	0.8109	
hesperidin		0.5271	0.5147	0.8121	
narirutin		0.8565	0.6025	0.3935	
eriodictyol		0.9270	0.0749	0.5811	increased
dihydrokaempferol		0.7532	0.7448	0.3671	
tetramethyl-o-scutellarin		0.0002	0.0129	0.0111	Increased
sinensetin		0.0007	0.0087	0.0257	Increased
chrysin		0.4411	0.3259	0.9234	
tangeritin		<.0001	0.0021	0.0296	Increased
diosmetin		<.0001	0.0312	0.0002	Increased
apigenin		0.4471	0.7958	0.4814	
chrysoeriol		<.0001	0.1565	0.0174	Increased
neoponcirin (isosakuranetin-7-rutinoside)		0.9438	0.4219	0.3742	
kaempferol		0.4542	0.9477	0.5915	
daidzein		0.0017	0.0032	0.0152	Decreased
genistein		0.0160	0.0284	0.0861	Decreased
glycitein		0.0010	0.0175	0.1517	Decreased
matairesinol		0.0296	0.3368	0.7654	Increased
secoisolariciresinol diglucoside		0.0912	0.3563	0.0839	increased
feruloylputrescine		0.2492	0.7445	0.0854	
ferulate		0.1613	0.3668	0.3879	
dihydroferulate		0.0583	0.3665	0.1185	increased
vanillate		0.0209	0.7656	0.0277	Increased
syringic acid		0.0646	0.7358	0.0548	
sinapate		0.9348	0.8990	0.4362	
tyrosol		0.8648	0.9802	0.2102	
coumestrol		0.1447	0.2431	0.3792	

Supplementary Table 3 - Fecal Metabolomics

		Day linear	Day quad	Day cubic	Change
Lysophospholipid	**Manova**	0.7858	0.7490	0.7849	
1-palmitoyl-GPC (16:0)		0.1892	0.2341	0.6263	
1-palmitoleoyl-GPC* (16:1)*		0.9000	0.0901	0.4822	
1-stearoyl-GPC (18:0)		0.1182	0.2540	0.3338	
1-oleoyl-GPC (18:1)		0.4110	0.1908	0.7605	
1-linoleoyl-GPC (18:2)		0.4603	0.4115	0.7641	
1-palmitoyl-GPE (16:0)		0.7761	0.6937	0.4445	
1-stearoyl-GPE (18:0)		0.2231	0.6562	0.3255	
1-oleoyl-GPE (18:1)		0.8845	0.8356	0.7360	
1-linoleoyl-GPE (18:2)*		0.4563	0.4733	0.9877	
1-stearoyl-GPS (18:0)*		0.1130	0.6688	0.5043	
1-palmitoyl-GPG (16:0)*		0.9823	0.3158	0.6052	
1-stearoyl-GPG (18:0)		0.1753	0.0749	0.3195	decreased
1-palmitoyl-GPI* (16:0)		0.7687	0.6059	0.1964	
1-stearoyl-GPI (18:0)		0.2601	0.5263	0.8163	
Phosphatidylcholine (PC)	**Manova**	0.0002	0.0014	0.3470	Decreased
1,2-dipalmitoyl-GPC (16:0/16:0)		0.0317	0.8171	0.7059	Decreased
1-palmitoyl-2-palmitoleoyl-GPC (16:0/16:1)*		0.0001	0.0064	0.6514	Decreased
1-palmitoyl-2-stearoyl-GPC (16:0/18:0)		0.0303	0.6701	0.3040	Decreased
1-palmitoyl-2-oleoyl-GPC (16:0/18:1)		0.0003	0.2130	0.8580	Decreased
1-palmitoyl-2-linoleoyl-GPC (16:0/18:2)		0.5464	0.5976	0.6478	
1-palmitoyl-2-arachidonoyl-GPC (16:0/20:4n6)		0.0002	0.3919	0.1917	Decreased
1-palmitoyl-2-docosahexaenoyl-GPC (16:0/22:6)		0.0055	0.0180	0.0221	Increased
1-stearoyl-2-oleoyl-GPC (18:0/18:1)		0.0023	0.3355	0.9297	Decreased
1-stearoyl-2-arachidonoyl-GPC (18:0/20:4)		0.0031	0.2188	0.2729	Decreased
1-oleoyl-2-linoleoyl-GPC (18:1/18:2)*		0.9601	0.8357	0.7076	
1,2-dilinoleoyl-GPC (18:2/18:2)		0.7142	0.9606	0.8690	
Phosphatidylethanolamine (PE)	**Manova**	0.1279	0.5445	0.9498	
1,2-dipalmitoyl-GPE (16:0/16:0)*		0.8151	0.6671	0.9788	
1-palmitoyl-2-oleoyl-GPE (16:0/18:1)		0.0514	0.3300	0.4630	
1-palmitoyl-2-linoleoyl-GPE (16:0/18:2)		0.8492	0.1256	0.5391	
1-palmitoyl-2-arachidonoyl-GPE (16:0/20:4)*		0.0151	0.3537	0.2300	Decreased
1-oleoyl-2-linoleoyl-GPE (18:1/18:2)*		0.4437	0.5869	0.4409	
1,2-dilinoleoyl-GPE (18:2/18:2)*		0.3530	0.5094	0.3250	
Phospholipid Metabolism	**Manova**	0.3737	0.4511	0.5760	
choline		0.6024	0.0819	0.3044	
phosphocholine		0.0468	0.9032	0.3677	Decreased
glycerophosphorylcholine (GPC)		0.2317	0.7533	0.6172	
glycerophosphoethanolamine		0.5912	0.2291	0.6443	
glycerophosphoserine*		0.6155	0.9778	0.1358	
glycerophosphoinositol*		0.9074	0.7115	0.8902	
trimethylamine N-oxide		0.1426	0.3391	0.3488	
**Polyamines**	**Manova**	0.0296	0.2271	0.7822	**Decreased**
agmatine		0.0102	0.1072	0.6748	Decreased
putrescine		0.0020	0.0210	0.0196	
N-acetyl-isoputreanine*		0.0007	0.0005	0.2591	Decreased
spermidine		<.0001	<.0001	0.0002	Decreased
diacetylspermidine*		0.3296	0.9868	0.1480	
N(1)-acetylspermine		0.0014	0.0069	0.0082	Decreased
N1,N12-diacetylspermine		0.0013	0.0016	0.0011	Decreased
5-methylthioadenosine (MTA)		0.0477	0.0284	0.2117	Decreased
N-acetylputrescine		0.0031	0.0495	0.1835	Decreased
4-acetamidobutanoate		0.2715	0.0272	0.2145	Decreased
(N(1) + N(8))-acetylspermidine		0.0035	0.0005	0.0011	Decreased
acetylagmatine		0.1096	0.0907	0.3752	
cadaverine		0.0083	0.0007	0.0153	Decreased
N-acetyl cadaverine		0.0091	0.0055	0.2411	Decreased
carboxyethyl-GABA		0.0059	0.0001	0.0169	Decreased
ornithine		0.0986	0.0980	0.8729	decreased

Supplementary Table 3 - Fecal Metabolomics

		Day linear	Day quad	Day cubic	Change
PostBiotics	**Manova**	<.0001	0.0169	0.0881	
2-keto-3-deoxy-gluconate		0.0716	0.0247	0.7003	Increased
2-piperidinone		0.0358	0.1812	0.0166	Decreased
3-dehydroshikimate		0.7602	0.9633	0.1879	
4-hydroxycinnamate		0.7471	0.6335	0.8299	
beta-guanidinopropanoate		0.0034	0.3474	0.0273	Decreased
diaminopimelate		0.0725	0.0049	0.0392	Decreased
enterodiol		0.1067	0.1522	0.5662	
enterolactone		0.2203	0.0386	0.0524	Decreased
equol		0.0306	0.5677	0.6250	Decreased
hesperetin		<.0001	0.0152	0.0001	Increased
histidine betaine (hercynine)*		0.3602	0.0935	0.7195	
levulinate (4-oxovalerate)		0.0005	0.3671	0.0159	Decreased
maltol		0.1157	0.1823	0.4136	
naringenin		0.0002	0.8756	0.0157	Increased
ponciretin		<.0001	0.0005	<.0001	Increased
rutinose		0.0001	0.1087	0.0045	Increased
secoisolariciresinol		0.0083	0.0108	0.0414	Increased
Urolithin A		0.0220	0.9383	0.9101	Increased
Primary Bile Acids	**Manova**	0.0612	0.4894	0.9896	decreased
cholate		0.0003	0.0455	0.2955	Decreased
glycocholate		0.0415	0.1381	0.1231	Decreased
taurocholate		0.9241	0.0975	0.2515	
chenodeoxycholate		0.0229	0.0348	0.3447	Decreased
glycochenodeoxycholate		0.0729	0.1549	0.3497	decreased
taurochenodeoxycholate		0.8779	0.1261	0.4399	
tauro-beta-muricholate		0.1631	0.1347	0.0717	decreased
7alpha-hydroxycholestenone		0.3929	0.1707	0.3380	
3b-hydroxy-5-cholenoic acid		0.1862	0.0055	0.3193	Decreased
7-hydroxycholesterol (alpha or beta)		0.9545	0.0140	0.2468	Decreased
Secondary Bile Acids	**Manova**	0.3648	0.8892	0.9159	
deoxycholate		0.9204	0.8344	0.3593	
3-dehydrodeoxycholate		0.0448	0.8934	0.3211	Increased
glycodeoxycholate		0.1597	0.0515	0.2370	
taurodeoxycholate		0.1942	0.2791	0.0474	Decreased
lithocholate		0.4110	0.9422	0.2417	
12-ketolithocholate		0.2401	0.2076	0.9382	
ursodeoxycholate		0.0613	0.1543	0.4200	decreased
isoursodeoxycholate		0.0276	0.1184	0.0990	Decreased
tauroursodeoxycholate		0.7270	0.1876	0.5691	
dehydrolithocholate		0.2556	0.4224	0.6621	
7,12-diketolithocholate		0.0145	0.8901	0.4246	Decreased
6-oxolithocholate		0.1661	0.1839	0.1422	
7-ketolithocholate		0.0073	0.0474	0.0328	Decreased
hyocholate		0.1267	0.1190	0.1295	
taurohyodeoxycholic acid		0.1051	0.2731	0.0727	decreased
glycohyodeoxycholate		0.8215	0.9740	0.9967	
3-dehydrocholate		0.0184	0.1034	0.1220	Decreased
12-dehydrocholate		0.0059	0.6612	0.5222	Decreased
7-ketodeoxycholate		0.0003	0.2950	0.6645	Decreased
ursocholate		0.4038	0.4045	0.9881	
isohyodeoxycholate		0.0952	0.0985	0.0919	decreased

Supplementary Table 3 - Fecal Metabolomics

		Day linear	Day quad	Day cubic	Change
Ceramides	**Manova**	0.0914	0.4170	0.7141	decreased
N-myristoyl-sphingosine (d18:1/14:0)*		0.0027	0.0052	0.0100	Decreased
N-palmitoyl-sphingosine (d18:1/16:0)		0.0018	0.0150	0.0042	Decreased
N-palmitoyl-phytosphingosine (t18:0/16:0)		0.0070	0.0112	0.0048	Decreased
N-(2-hydroxypalmitoyl)-sphingosine (d18:1/16:0(2OH))		0.0719	0.2520	0.0064	Decreased
N-stearoyl-sphingosine (d18:1/18:0)*		0.0002	0.0016	0.0074	Decreased
N-oleoyl-sphingosine (d18:1/18:1)*		0.0012	0.0161	0.0120	Decreased
ceramide (d18:1/17:0, d17:1/18:0)*		0.0011	0.0039	0.0017	Decreased
ceramide (d18:1/20:0, d16:1/22:0, d20:1/18:0)*		0.0001	0.0013	0.0008	Decreased
ceramide (d18:2/24:1, d18:1/24:2)*		0.1338	0.2750	0.0518	
Dihydroceramides	**Manova**	0.0104	0.0552	0.2416	Decreased
N-palmitoyl-sphinganine (d18:0/16:0)		0.0005	0.0014	0.0010	Decreased
N-stearoyl-sphinganine (d18:0/18:0)*		0.0001	<.0001	0.0003	Decreased
Dihydrosphingomyelins	**Manova**	0.0442	0.2683	0.2537	**Decreased**
palmitoyl dihydrosphingomyelin (d18:0/16:0)*		0.0146	0.0331	0.0068	Decreased
sphingomyelin (d18:0/18:0, d19:0/17:0)*		0.0021	0.0142	0.0044	Decreased
Hexosylceramides (HCER)	**Manova**	0.0156	0.1452	0.6551	**Decreased**
glycosyl-N-palmitoyl-sphingosine (d18:1/16:0)		0.0934	0.0227	0.3563	decreased
glycosyl-N-stearoyl-sphingosine (d18:1/18:0)		0.0054	0.0003	0.0045	Decreased
glycosyl-N-behenoyl-sphingosine (d18:1/22:0)*		0.0516	0.0096	0.0221	decreased
glycosyl-N-(2-hydroxynervonoyl)-sphingosine (d18:1/24:1(2OH))*		0.0479	0.0099	0.9720	Decreased
glycosyl ceramide (d18:1/20:0, d16:1/22:0)*		0.0012	0.0016	0.0266	Decreased
Sphingolipid Synthesis	**Manova**	0.0171	0.0651	0.2027	Decreased
3-ketosphinganine		0.0148	0.0244	0.0162	Decreased
sphinganine		0.0002	0.0003	0.0005	Decreased
sphingadienine		<.0001	<.0001	0.0001	Decreased
phytosphingosine		0.0199	0.0263	0.0279	Decreased
Sphingosines	**Manova**	0.1122	0.3652	0.6858	
sphingosine		<.0001	0.0056	0.0053	Decreased
N-acetylsphingosine		0.0601	0.0932	0.2062	
hexadecasphingosine (d16:1)*		0.0204	0.0094	0.0447	Decreased
heptadecasphingosine (d17:1)		0.0013	0.0074	0.0038	Decreased
eicosanoylsphingosine (d20:1)*		0.0002	0.0147	0.0069	Decreased
dehydrophytosphingosine*		0.0018	<.0001	0.0333	Decreased
N-stearoyl-phytosphingosine (t18:0/18:0)*		0.0563	0.0595	0.0475	decreased
Sphingomyelins	**Manova**	0.0091	0.2015	0.7950	**Decreased**
palmitoyl sphingomyelin (d18:1/16:0)		0.1035	0.1499	0.0254	Decreased
hydroxypalmitoyl sphingomyelin (d18:1/16:0(OH))**		0.0173	0.0730	0.0055	Decreased
stearoyl sphingomyelin (d18:1/18:0)		0.0146	0.0639	0.0061	Decreased
behenoyl sphingomyelin (d18:1/22:0)*		0.1158	0.1444	0.0219	Decreased
tricosanoyl sphingomyelin (d18:1/23:0)*		0.5958	0.3283	0.0698	
lignoceroyl sphingomyelin (d18:1/24:0)		0.2454	0.2687	0.0314	Decreased
sphingomyelin (d18:1/17:0, d17:1/18:0, d19:1/16:0)		0.0200	0.0130	0.0028	Decreased
sphingomyelin (d18:1/20:0, d16:1/22:0)*		0.0378	0.0515	0.0073	Decreased
sphingomyelin (d18:1/24:1, d18:2/24:0)*		0.0260	0.0745	0.0449	Decreased
sphingomyelin (d18:2/24:1, d18:1/24:2)*		0.2304	0.2481	0.0320	Decreased

Supplementary Table 3 - Fecal Metabolomics

		Day linear	Day quad	Day cubic	Change
Terpenoids	**Manova**	0.0004	0.1870	0.4491	Increased
pheophytin A		0.2788	0.9172	0.2161	
pheophorbide A		0.3723	0.2999	0.0677	
limonin		0.0008	0.0982	0.2490	Increased
nomilin		0.0044	0.0460	0.2084	Increased
oleanolate		0.0646	0.0513	0.0027	Increased
lanosterol		0.0014	0.0007	0.0074	Decreased
beta-sitosterol		0.0461	0.4606	0.3702	Increased
stigmasterol		0.0782	0.8966	0.3130	
stigmastadienone		0.0011	0.0229	0.0507	Increased
campesterol		0.7799	0.4378	0.5254	
fucosterol		0.1405	0.4414	0.3361	
ergosterol		0.1506	0.7410	0.0023	Increased
carotene diol (1)		0.0100	0.1058	0.4501	Increased
carotene diol (2)		0.9403	0.7486	0.9434	
carotene diol (3)		0.0512	0.0842	0.0813	increased
Tocopherol Metabolism	**Manova**	0.0002	0.0351	0.9341	
alpha-tocopherol		0.1015	0.7893	0.2706	
alpha-tocopherol acetate		0.0356	0.0328	0.8833	Increased
delta-tocopherol		0.0852	0.0525	0.6701	decreased
alpha-tocotrienol		0.1109	0.6085	0.1579	
gamma-tocotrienol		0.0339	0.3634	0.6194	Decreased
gamma-CEHC		0.1749	0.1909	0.4382	
alpha-CEHC sulfate		0.0182	0.0395	0.5762	Increased
alpha-CEHC taurine*		0.0007	0.0055	0.6871	Increased
alpha-CEHC		0.0199	0.0214	0.2279	Increased
delta-CEHC		0.1842	0.1547	0.3103	
gamma-CEHC sulfate*		0.2386	0.5499	0.7514	
gamma-CEHC taurine*		0.9232	0.5502	0.8988	
gamma-tocopherol/beta-tocopherol		0.0475	0.0002	0.2490	Decreased
Indole Pathway	**Manova**	0.0003	0.3071	0.4532	
indole		0.0155	0.0276	0.1372	Decreased
3-indoxyl sulfate		0.1575	0.0311	0.8749	Decreased
indolelactate		0.8615	0.7038	0.2927	
indolepropionate		0.3084	0.6766	0.7860	
tryptamine		0.1607	0.6447	0.3389	
indoleacetate		0.0190	0.8282	0.2215	Increased
indoleacrylate		0.6483	0.4027	0.1241	
indole-3-carboxylate		0.0010	0.3269	0.0753	Increased
2-oxindole-3-acetate		0.0133	0.0082	0.1096	Increased
indoleacetylglutamine		0.3003	0.8586	0.5005	
indoleacetylglycine		0.2504	0.0928	0.4582	
3-formylindole		0.3852	0.0603	0.5449	
methyl indole-3-acetate		0.2175	0.3120	0.3609	
3-hydroxyindolin-2-one		0.0607	0.3411	0.6570	
indolin-2-one		0.0016	0.0024	0.0018	Decreased
Kynurenine Pathway	**Manova**	0.1734	0.4871	0.8969	
N-formylanthranilic acid		0.0043	0.0065	0.0418	Decreased
kynurenine		0.7948	0.4877	0.7403	
kynurenate		0.2069	0.9367	0.7145	
anthranilate		0.6762	0.0932	0.9103	
2-aminophenol		0.0122	0.5289	0.4851	Increased
picolinate		0.0447	0.0031	0.0087	Decreased
quinolinate		0.9683	0.8143	0.8956	
Serotonin Pathway	**Manova**	0.0484	0.5940	0.4209	Decreased
serotonin		0.0005	0.1539	0.0352	Decreased
5-hydroxyindoleacetate		0.0309	0.3785	0.8274	

P values <0.05 are shaded in yellow; P values ≥0.05 to <0.10 are shaded in blue.
