# Supplementary material for: Microbiome function underpins the efficacy of a fiber-supplemented dietary intervention in dogs with chronic large bowel diarrhea
Source: BMC Vet Res. 2022 Jun 24;18:245. doi: 10.1186/s12917-022-03315-3 (PMC9233311; doi:10.1186/s12917-022-03315-3)
Supplement: Supplementary file 4 — Additional file 4. [file 12917_2022_3315_MOESM4_ESM.pdf]

Supplementary Table 4 - Serum Metabolomics

		Day linear	Day Quad	Day Cubic	Change
Amino Acids	**Manova**	0.0002	0.8057	<.0001	**Increased**
alanine		0.3255	0.4768	0.0253	
arginine		0.2914	0.1555	0.1754	
asparagine		0.0134	0.0286	<.0001	Increased
aspartate		0.7447	0.7376	0.1990	
cysteine		0.1319	0.1767	0.3116	
glutamate		0.6999	0.7863	0.0976	decreased
glutamine		0.1227	0.2300	0.1642	
glycine		0.2374	0.9495	0.0115	
histidine		0.2684	0.4088	0.1815	
isoleucine		0.0554	0.4920	0.9874	decreased
leucine		0.5224	0.1336	<.0001	Increased
lysine		0.9541	0.0201	0.0006	Increased
methionine		<.0001	0.0101	0.0002	
phenylalanine		0.3196	0.2644	0.5061	
proline		0.0006	0.2133	0.0146	Increased
serine		0.1748	0.5389	0.1414	
taurine		0.0012	0.0132	0.5565	Increased
threonine		<.0001	0.0656	0.5715	Increased
tryptophan		0.0203	0.0122	0.0569	Increased
tyrosine		0.0217	0.3929	0.0863	Increased
valine		0.2817	0.8044	0.3131	
Carbohydrate Metabolism	**Manova**	0.0004	0.3136	0.0023	**Increased**
arabitol/xylitol		0.0033	0.2036	0.0148	Increased
arabonate/xylonate		<.0001	0.0141	0.3052	Increased
erythritol		0.0081	0.3602	0.3754	Increased
erythronate*		0.0017	0.5783	0.3130	Increased
fructose		0.4310	0.2503	0.5708	
galactonate		0.0192	0.1757	0.9365	Increased
glucose		0.3890	0.2941	0.5761	
glucuronate		0.7993	0.2494	0.7596	
glycerate		0.0139	0.0447	0.5387	Increased
lactate		0.3226	0.0793	0.1003	
mannitol/sorbitol		0.5809	0.7610	0.0482	Increased
mannonate*		0.0203	0.1186	0.0083	
mannose		0.0035	0.1118	0.1213	Decreased
pyruvate		0.2181	0.3410	0.1892	
ribitol		0.0237	0.7859	0.4364	Increased
ribonate		0.4695	0.8705	0.6907	
ribose		0.0423	0.3162	0.1757	Increased
ribulonate/xylulonate/lyxonate*		<.0001	0.0216	<.0001	Increased
Collagen Metabolism	**Manova**	0.618	0.8433	<.0001	**Decreased**
5-hydroxylysine		0.5981	0.4884	0.361	
hydroxyproline		0.3266	0.6868	0.0023	Decreased
prolylhydroxyproline		0.1963	0.7391	0.4948	
Eicosanoids	**Manova**	0.0136	0.7214	0.4539	**Decreased**
thromboxane B2		0.0709	0.4522	0.2430	decreased
prostaglandin F2alpha		0.0884	0.1986	0.5287	decreased
5-HEPE		0.6781	0.3338	0.8376	
5-HETE		0.5252	0.7737	0.8784	
12-HETE		0.6896	0.7270	0.0040	Decreased
12-HHTrE		0.0792	0.5699	0.2169	decreased

Supplementary Table 4 - Serum Metabolomics

		Day linear	Day Quad	Day Cubic	Change
Polyunsaturated n3 Fatty Acids	**Manova**	<.0001	0.0624	0.6511	**Increased**
linolenate (18:3n3 or 3n6)		0.0024	0.0839	0.4578	Increased
hexadecatrienoate (16:3n3)		0.0128	0.2194	0.8634	Increased
stearidonate (18:4n3)		<.0001	0.0378	0.3108	Increased
eicosapentaenoate (EPA; 20:5n3)		<.0001	0.1030	0.6907	Increased
heneicosapentaenoate (21:5n3)		<.0001	<.0001	0.3976	Increased
docosatrienoate (22:3n3)		0.7253	0.1459	0.5158	
docosapentaenoate (DPA; 22:5n3)		<.0001	0.0007	0.5509	Increased
docosahexaenoate (DHA; 22:6n3)		<.0001	0.0007	0.0939	Increased
Polyunsaturated n6 Fatty Acids	**Manova**	<.0001	0.1957	0.4774	**Decreased**
hexadecadienoate (16:2n6)		0.8385	0.7053	0.3501	
linoleate (18:2n6)		0.2771	0.5107	0.6294	
dihomolinoleate (20:2n6)		0.5069	0.1495	0.3800	
arachidonate (20:4n6)		0.1479	0.0822	0.4143	
docosadienoate (22:2n6)		0.0384	0.6017	0.2984	Decreased
docosatrienoate (22:3n6)*		<.0001	0.0011	0.0698	Decreased
adrenate (22:4n6)		<.0001	0.0024	0.6700	Decreased
docosapentaenoate (n6 DPA; 22:5n6)		<.0001	0.0148	0.3732	Decreased
Short, Medium, and Long Chain Fatty Acids	**Manova**	<.0001	0.3616	0.159	**Decreased**
butyrate (4:0)		0.0047	0.3787	0.207	Increased
valerate (5:0)		0.7002	0.0457	0.4064	
caproate (6:0)		0.4828	0.0136	0.2348	Decreased
heptanoate (7:0)		0.4641	0.4988	0.3209	
caprylate (8:0)		0.4987	0.1096	0.0435	Decreased
pelargonate (9:0)		0.6064	0.4166	0.808	
caprate (10:0)		0.3374	0.2863	0.9975	
cis-4-decenoate (10:1n6)*		0.7634	0.1458	0.6583	
(2 or 3)-decenoate (10:1n7 or n8)		0.8252	0.9367	0.207	
10-undecenoate (11:1n1)		0.4411	0.6335	0.2177	
laurate (12:0)		0.4493	0.9211	0.5426	
5-dodecenoate (12:1n7)		0.2828	0.2202	0.6318	
myristate (14:0)		0.9948	0.504	0.3215	
myristoleate (14:1n5)		0.4655	0.3623	0.4932	
pentadecanoate (15:0)		0.2247	0.1374	0.5949	
palmitate (16:0)		0.1987	0.8386	0.7195	
palmitoleate (16:1n7)		0.2437	0.847	0.348	
margarate (17:0)		0.5917	0.2325	0.1439	
10-heptadecenoate (17:1n7)		0.0478	0.1263	0.1386	Decreased
stearate (18:0)		0.6188	0.4398	0.0829	
oleate/vaccenate (18:1)		0.1304	0.7856	0.1316	
nonadecanoate (19:0)		0.0055	0.0081	0.2548	
10-nonadecenoate (19:1n9)		0.4918	0.4321	0.4294	
arachidate (20:0)		0.6702	0.2681	0.2167	
behenate (22:0)*		0.0115	0.0096	0.8616	Increased
erucate (22:1n9)		0.5768	0.9252	0.3948	
nervonate (24:1n9)*		0.2468	0.1574	0.2779	

Supplementary Table 4 - Serum Metabolomics

		Day linear	Day Quad	Day Cubic	Change
Lysophospholipid	**Manova**	<.0001	0.008	<.0001	
1-palmitoyl-GPA (16:0)		0.232	0.6743	0.1088	
1-linoleoyl-GPA (18:2)*		<.0001	0.0126	0.0005	Increased
1-linolenoyl-GPA (18:3)*		<.0001	0.0112	0.0532	Increased
1-arachidonoyl-GPA (20:4)		0.0007	0.0157	0.431	Decreased
1-palmitoyl-GPC (16:0)		0.7874	0.8778	0.1146	
1-palmitoleoyl-GPC (16:1)*		0.232	0.6743	0.1088	
1-stearoyl-GPC (18:0)		0.0009	0.0043	0.1192	Increased
1-oleoyl-GPC (18:1)		0.0089	0.1809	0.7455	Increased
1-linoleoyl-GPC (18:2)		0.2319	0.5852	0.775	
1-linolenoyl-GPC (18:3)*		0.0095	0.0713	0.0029	Increased
1-arachidonoyl-GPC (20:4n6)*		0.0092	0.589	0.1738	
1-lignoceroyl-GPC (24:0)		<.0001	0.0968	0.6486	Increased
1-cerotoyl-GPC (26:0)*		<.0001	0.2531	0.0768	Decreased
1-palmitoyl-GPE (16:0)		0.3414	0.0975	0.0553	
1-stearoyl-GPE (18:0)		0.0127	0.0651	0.0623	Increased
2-stearoyl-GPE (18:0)*		0.4554	0.0174	0.4387	
1-oleoyl-GPE (18:1)		0.0797	0.4171	0.9284	
1-linoleoyl-GPE (18:2)*		0.0002	0.1165	0.005	Increased
1-arachidonoyl-GPE (20:4n6)*		0.7281	0.9975	0.6295	
1-stearoyl-GPS (18:0)*		0.9491	0.09	0.9676	
1-oleoyl-GPS (18:1)		0.6304	0.1997	0.2246	
1-linoleoyl-GPS (18:2)*		0.5681	0.0544	0.1848	
1-palmitoyl-GPG (16:0)*		0.1696	0.0522	0.0221	Increased
1-stearoyl-GPG (18:0)		0.2872	0.2764	0.4893	
1-linoleoyl-GPG (18:2)*		0.0466	0.0518	0.0607	Increased
1-palmitoyl-GPI (16:0)		0.0009	0.0043	0.1192	
1-stearoyl-GPI (18:0)		0.5174	0.2348	0.2394	
1-oleoyl-GPI (18:1)		0.7104	0.4787	0.1416	
1-linoleoyl-GPI (18:2)*		0.5681	0.0544	0.1848	
1-arachidonoyl-GPI (20:4)*		<.0001	0.2531	0.0768	
Phosphatidylcholine (PC)	**Manova**	<.0001	0.0399	<.0001	**Increased**
1,2-dilinolenoyl-GPC (18:3/18:3)*		<.0001	0.0212	<.0001	Increased
1,2-dilinoleoyl-GPC (18:2/18:2)		<.0001	0.1783	0.1173	Increased
1,2-dipalmitoyl-GPC (16:0/16:0)		<.0001	0.0619	0.6191	Increased
1-linoleoyl-2-linolenoyl-GPC (18:2/18:3)*		<.0001	0.0124	<.0001	Increased
1-myristoyl-2-arachidonoyl-GPC (14:0/20:4)*		0.9785	0.8199	0.015	Increased
1-myristoyl-2-palmitoyl-GPC (14:0/16:0)		0.3639	0.4479	0.0098	
1-oleoyl-2-docosahexaenoyl-GPC (18:1/22:6)*		<.0001	0.0001	0.008	Increased
1-oleoyl-2-linoleoyl-GPC (18:1/18:2)*		0.0862	0.6218	0.5021	
1-palmitoleoyl-2-linolenoyl-GPC (16:1/18:3)*		<.0001	0.1899	0.0012	Increased
1-palmitoleoyl-2-linoleoyl-GPC (16:1/18:2)*		0.8588	0.9072	0.4449	
1-palmitoyl-2-arachidonoyl-GPC (16:0/20:4n6)		<.0001	0.0106	0.8353	Decreased
1-palmitoyl-2-docosahexaenoyl-GPC (16:0/22:6)		<.0001	<.0001	<.0001	Increased
1-palmitoyl-2-linoleoyl-GPC (16:0/18:2)		0.2786	0.1656	0.3615	
1-palmitoyl-2-oleoyl-GPC (16:0/18:1)		0.0023	0.0096	0.0037	Decreased
1-palmitoyl-2-palmitoleoyl-GPC (16:0/16:1)*		0.0004	0.0003	0.0053	Decreased
1-palmitoyl-2-stearoyl-GPC (16:0/18:0)		0.0105	0.0979	0.0749	Increased
1-stearoyl-2-arachidonoyl-GPC (18:0/20:4)		0.0064	0.3759	0.0245	
1-stearoyl-2-docosahexaenoyl-GPC (18:0/22:6)		<.0001	<.0001	<.0001	Increased
1-stearoyl-2-linoleoyl-GPC (18:0/18:2)*		<.0001	0.0701	0.1209	Increased
1-stearoyl-2-oleoyl-GPC (18:0/18:1)		0.0018	0.0525	0.3321	Decreased

Supplementary Table 4 - Serum Metabolomics

		Day linear	Day Quad	Day Cubic	Change
Phosphatidylethanolamine (PE)	**Manova**	<.0001	0.0399	0.0054	**Decreased**
1-palmitoyl-2-oleoyl-GPE (16:0/18:1)		0.0788	0.1609	0.0914	
1-palmitoyl-2-linoleoyl-GPE (16:0/18:2)		0.7352	0.618	0.4406	
1-palmitoyl-2-arachidonoyl-GPE (16:0/20:4)*		0.0707	0.1957	0.281	
1-palmitoyl-2-docosahexaenoyl-GPE (16:0/22:6)*		0.0113	0.0844	0.0799	Increased
1-stearoyl-2-oleoyl-GPE (18:0/18:1)		0.4495	0.4015	0.2958	
1-stearoyl-2-linoleoyl-GPE (18:0/18:2)*		0.0883	0.1927	0.1469	
1-stearoyl-2-arachidonoyl-GPE (18:0/20:4)		0.0804	0.8032	0.1106	
1-stearoyl-2-docosahexaenoyl-GPE (18:0/22:6)*		<.0001	0.0064	0.0011	Increased
1,2-dioleoyl-GPE (18:1/18:1)		0.3886	0.379	0.1686	
1-oleoyl-2-linoleoyl-GPE (18:1/18:2)*		0.2265	0.5373	0.712	
Phosphatidylinositol (PI)	**Manova**	<.0001	0.1991	0.0516	**Increased**
1-palmitoyl-2-oleoyl-GPI (16:0/18:1)*		0.2764	0.3305	0.1082	
1-palmitoyl-2-linoleoyl-GPI (16:0/18:2)		0.0272	0.1697	0.0158	Increased
1-palmitoyl-2-arachidonoyl-GPI (16:0/20:4)*		0.3323	0.6131	0.1548	
1-stearoyl-2-oleoyl-GPI (18:0/18:1)*		0.0908	0.1531	0.035	Decreased
1-stearoyl-2-linoleoyl-GPI (18:0/18:2)		<.0001	0.0981	0.2978	Increased
1-stearoyl-2-arachidonoyl-GPI (18:0/20:4)		0.7538	0.9309	0.8789	
Ceramides	**Manova**	<.0001	0.4337	0.0125	**Decreased**
ceramide (d16:1/24:1, d18:1/22:1)*		0.4316	0.2738	0.2564	
ceramide (d18:1/17:0, d17:1/18:0)*		0.2753	0.2989	0.3213	
ceramide (d18:2/24:1, d18:1/24:2)*		0.5995	0.4248	0.3247	
N-behenoyl-sphingadienine (d18:2/22:0)*		0.9062	0.3713	0.2801	
N-palmitoyl-heptadecasphingosine (d17:1/16:0)*		0.3416	0.3199	0.3207	
N-palmitoyl-sphingosine (d18:1/16:0)		0.4200	0.3960	0.3698	
N-stearoyl-sphingadienine (d18:2/18:0)*		0.2141	0.2345	0.2435	
N-stearoyl-sphingosine (d18:1/18:0)*		0.2544	0.3025	0.4314	
Dihydroceramides	**Manova**	0.0298	0.7896	0.1297	
N-palmitoyl-sphinganine (d18:0/16:0)		0.3660	0.2807	0.3517	
N-arachidoyl-sphingosine (d18:1/20:0)*		0.5662	0.2115	0.2537	
Dihydrosphingomyelins	**Manova**	<.0001	0.3523	0.4449	**Decreased**
behenoyl dihydrosphingomyelin (d18:0/22:0)*		0.1417	0.8998	0.222	
myristoyl dihydrosphingomyelin (d18:0/14:0)*		0.0300	0.5419	0.5985	Increased
palmitoyl dihydrosphingomyelin (d18:0/16:0)*		0.4677	0.7127	0.5237	
sphingomyelin (d18:0/18:0, d19:0/17:0)*		<.0001	0.0009	0.5737	Decreased
sphingomyelin (d18:0/20:0, d16:0/22:0)*		<.0001	0.0034	0.6821	Decreased
Hexosylceramides (HCER)	**Manova**	0.0071	0.649	0.1742	
glycosyl ceramide (d18:1/20:0, d16:1/22:0)*		0.0539	0.6545	0.2381	
glycosyl ceramide (d18:2/24:1, d18:1/24:2)*		0.2953	0.4588	0.2581	
glycosyl-N-(2-hydroxynervonoyl)-sphingosine (d18:1/24:1(2OH))*		0.1788	0.2646	0.7527	
glycosyl-N-stearoyl-sphingosine (d18:1/18:0)		0.0748	0.6529	0.1000	
Lactosylceramides (LCER)	**Manova**	0.1662	0.4969	0.0159	
lactosyl-N-palmitoyl-sphingosine (d18:1/16:0)		0.0151	0.2850	0.0037	Increased
lactosyl-N-stearoyl-sphingosine (d18:1/18:0)*		0.6205	0.6222	0.3562	
Sphingolipid Synthesis	**Manova**	0.4804	0.9919	0.2069	
sphinganine		0.1693	0.6716	0.1839	
sphinganine-1-phosphate		0.7376	0.4879	0.3674	
Sphingosines	**Manova**	0.6508	0.8421	0.404	
sphingosine		0.4365	0.7822	0.2833	
sphingosine 1-phosphate		0.8069	0.1735	0.8355	

Supplementary Table 4 - Serum Metabolomics

		Day linear	Day Quad	Day Cubic	Change
Sphingomyelins	**Manova**	<.0001	0.0513	0.0283	**Increased**
behenoyl sphingomyelin (d18:1/22:0)*		0.1348	0.4844	0.6621	
lignoceroyl sphingomyelin (d18:1/24:0)		0.4555	0.3595	0.0269	Decreased
palmitoyl sphingomyelin (d18:1/16:0)		0.0488	0.0860	0.4263	Increased
sphingomyelin (d17:1/14:0, d16:1/15:0)*		<.0001	0.0015	0.0268	Decreased
sphingomyelin (d17:1/16:0, d18:1/15:0, d16:1/17:0)*		0.0294	0.2885	0.8175	Increased
sphingomyelin (d17:2/16:0, d18:2/15:0)*		0.1746	0.3148	0.9970	
sphingomyelin (d18:1/14:0, d16:1/16:0)*		0.0067	0.1663	0.0031	Increased
sphingomyelin (d18:1/17:0, d17:1/18:0, d19:1/16:0)		0.0305	0.0143	0.0081	Increased
sphingomyelin (d18:1/18:1, d18:2/18:0)		0.0137	0.0967	0.3543	Increased
sphingomyelin (d18:1/19:0, d19:1/18:0)*		0.2635	0.3616	0.9990	
sphingomyelin (d18:1/20:0, d16:1/22:0)*		0.0030	0.1692	0.7547	Increased
sphingomyelin (d18:1/20:1, d18:2/20:0)*		0.0205	0.6532	0.0513	Decreased
sphingomyelin (d18:1/20:2, d18:2/20:1, d16:1/22:2)*		<.0001	0.0002	0.5168	Increased
sphingomyelin (d18:1/21:0, d17:1/22:0, d16:1/23:0)*		0.0061	0.0798	0.6619	Increased
sphingomyelin (d18:1/22:1, d18:2/22:0, d16:1/24:1)*		0.1207	0.7741	0.7881	
sphingomyelin (d18:1/22:2, d18:2/22:1, d16:1/24:2)*		0.5611	0.2249	0.0196	
sphingomyelin (d18:1/24:1, d18:2/24:0)*		0.0014	0.0423	0.9527	Increased
sphingomyelin (d18:1/25:0, d19:0/24:1, d20:1/23:0, d19:1/24:0)*		0.2454	0.2188	0.6589	
sphingomyelin (d18:2/14:0, d18:1/14:1)*		0.1524	0.0250	0.9391	Increased
sphingomyelin (d18:2/16:0, d18:1/16:1)*		<.0001	0.0003	0.0351	Decreased
sphingomyelin (d18:2/18:1)*		0.3379	0.6105	0.0701	
sphingomyelin (d18:2/21:0, d16:2/23:0)*		0.0003	0.0197	0.6110	Increased
sphingomyelin (d18:2/23:0, d18:1/23:1, d17:1/24:1)*		0.0135	0.4188	0.2573	Decreased
sphingomyelin (d18:2/23:1)*		0.0016	0.0308	0.2785	Decreased
sphingomyelin (d18:2/24:1, d18:1/24:2)*		<.0001	0.0003	0.3974	Increased
sphingomyelin (d18:2/24:2)*		0.0159	0.2733	0.4202	Decreased
stearoyl sphingomyelin (d18:1/18:0)		0.0121	0.0709	0.9236	Increased
tricosanoyl sphingomyelin (d18:1/23:0)*		0.6592	0.8965	0.9908	
Tocopherol Metabolism	**Manova**	<.0001	0.004	0.0017	**Increased**
alpha-tocopherol		<.0001	0.0162	<.0001	Increased
alpha-tocopherol acetate		0.4727	0.6465	0.0018	Increased
gamma-tocopherol/beta-tocopherol		0.0003	0.0013	0.2204	Decreased
alpha-CEHC glucuronide*		<.0001	<.0001	0.0014	Increased
alpha-CEHC sulfate		<.0001	<.0001	<.0001	Increased
alpha-CEHC		0.6321	0.2210	0.1405	
gamma-CEHC sulfate		0.7901	0.6714	0.6740	
Tryptophan Indole Pathway	**Manova**	0.1342	0.0298	0.0192	
indolepropionate		0.2160	0.1347	0.0064	Increased
indolelactate		0.5286	0.0341	0.2420	
indoleacrylate		0.2314	0.0018	0.2252	Increased
indoleacetate		0.7700	0.0596	0.0045	Increased
methyl indole-3-acetate		0.4359	0.7658	0.3057	
indoleacetylglutamine		0.4347	0.0783	0.6380	increased
indoleacetylglycine		0.5775	0.0625	0.0521	increased
indoleacetylalanine		0.3412	0.3955	0.0038	Increased
2-oxindole-3-acetate		0.0270	0.0170	0.0103	Increased
3-indoleglyoxylic acid		0.2381	0.0216	0.7752	Increased
3-formylindole		0.6346	0.2971	0.3871	
indolin-2-one		0.4501	0.1668	0.5058	
3-indoxyl sulfate		0.5051	0.1545	0.6453	
5-hydroxyindole sulfate		0.1010	0.0451	0.6605	Decreased
7-hydroxyindole sulfate		0.0928	0.4311	0.9842	decreased
indoxyl glucuronide		0.3775	0.1052	0.4437	

P values <0.05 are shaded in yellow; P values ≥0.05 to <0.10 are shaded in blue.
